# Supplementary material for: A phase III double-blind, placebo-controlled, randomized withdrawal trial of 5‑aminolevulinic acid hydrochloride with sodium ferrous citrate for efficacy and safety in patients diagnosed as Leigh syndrome
Source: PLoS One. 2026 Jul 17;21(7):e0332283. doi: 10.1371/journal.pone.0332283 (PMC13379092; doi:10.1371/journal.pone.0332283)
Supplement: S1 Table — (DOCX) [file pone.0332283.s001.docx]

**S1 Table. Dosage and administration of Investigational drug**

| Body weight* | Daily dose  (5-ALA/SFC) | Number of doses and 5-ALA, SFC per dose  Number of capsules taken for each |
| --- | --- | --- |
| >20 kg | 50 mg/78.44 mg | Twice a day, 1 capsule in the morning and  1 capsule in the evening |
| ≥ 20 kg and <30 kg | 75 mg/117.66 mg | Twice a day, 2 capsules in the morning and  1 capsule in the evening |
| ≥ 30 kg and <40 kg | 100 mg/156.88 mg | Twice a day, 2 capsules in the morning and  2 capsules in the evening |
| ≥ 40 kg and <50 kg | 125 mg/196.1 mg | Twice a day, 3 capsules in the morning and  2 capsules in the evening |
| ≥ 50 kg | 150 mg/235.32 mg | Twice a day, 3 capsules in the morning and  3 capsules in the evening |

*: Baseline body weight during the observation period.
