# Supplementary material for: A phase III double-blind, placebo-controlled, randomized withdrawal trial of 5‑aminolevulinic acid hydrochloride with sodium ferrous citrate for efficacy and safety in patients diagnosed as Leigh syndrome
Source: PLoS One. 2026 Jul 17;21(7):e0332283. doi: 10.1371/journal.pone.0332283 (PMC13379092; doi:10.1371/journal.pone.0332283)
Supplement: S2 Table — (DOCX) [file pone.0332283.s002.docx]

**S2 Table. Change from baseline: NPMDS score (cranial nervous symptoms and myopathy symptoms), mean ±S.D. (LOCF, FAS）**

|  | Open-label period discontinued  (*n*=26) | DB-period  SPP-004 (*n* =13) | DB-period  Placebo (*n* =14) |
| --- | --- | --- | --- |
| Open-label period 0-Week | 0.0 ± 0.00 | 0.0 ± 0.00 | 0.0 ± 0.00 |
| 12-Week | -0.2 ± 1.22 | -0.4 ± 1.02 | -1.1 ± 1.44 |
| 24-Week / DB 0-Week | 0.1 ± 0.43 | -1.6 ± 1.82 | -1.9 ± 1.00 |
| DB 4-Week | - | -2.0 ± 2.31 | -1.5 ± 1.16 |
| DB 8-Week | - | -1.8 ± 2.34 | -1.5 ± 0.94 |
| DB 12-Week | - | -2.2 ± 1.95 | -1.0 ± 1.75 |
| DB 16-Week | - | -2.2 ± 2.12 | -1.0 ± 1.75 |
| DB 20-Week | - | -2.2 ± 2.09 | -1.0 ± 1.75 |
| DB 24-Week | - | -2.2 ± 2.12 | -1.0 ± 1.75 |
| DB 28-Week | - | -2.3 ± 2.39 | -1.0 ± 1.75 |
| DB 32-Week | - | -2.3 ± 2.39 | -1.0 ± 1.75 |
| DB 36-Week | - | -2.3 ± 2.32 | -1.1 ± 1.83 |
| DB 40-Week | - | -2.3 ± 2.32 | -1.1 ± 1.83 |
| DB 44-Week | - | -2.3 ± 2.32 | -1.1 ± 1.83 |
| DB 48-Week | - | -2.3 ± 2.18 | -1.1 ± 1.83 |
