# Supplementary material for: A phase III double-blind, placebo-controlled, randomized withdrawal trial of 5‑aminolevulinic acid hydrochloride with sodium ferrous citrate for efficacy and safety in patients diagnosed as Leigh syndrome
Source: PLoS One. 2026 Jul 17;21(7):e0332283. doi: 10.1371/journal.pone.0332283 (PMC13379092; doi:10.1371/journal.pone.0332283)
Supplement: S3 Table — (DOCX) [file pone.0332283.s003.docx]

**S3 Table. Change in full NPMDS score (Section I-IV), mean ±S.D. (LOCF, FAS）**

|  | Open-label period discontinued  (*n*=26) | DB-period  SPP-004 (*n* =13) | DB-period  Placebo (*n* =14) |
| --- | --- | --- | --- |
| **Absolute Values** |  |  |  |
| Open-label period 0-Week | 52.4 ± 18.0 | 50.3 ± 20.1 | 40.9 ± 19.4 |
| 12-Week | 50.4 ± 16.8 | 47.9 ± 17.5 | 38.0 ± 19.6 |
| 24-Week / DB 0-Week | 50.1 ± 16.4 | 46.3 ± 17.9 | 37.2 ± 19.3 |
| DB 12-Week |  | 43.6 ± 16.5 | 39.3 ± 20.3 |
| DB 24-Week |  | 45.1 ± 16.4 | 39.2 ± 20.1 |
| DB 36-Week |  | 44.3 ± 16.7 | 39.0 ± 20.5 |
| DB 48-Week |  | 45.4 ± 16.4 | 38.9 ± 20.1 |
|  |  |  |  |
| **Relative change from baseline** |  |  |  |
| Open-label period 0-Week | 0.0 ± 0.0 | 0.0 ± 0.0 | 0.0 ± 0.0 |
| 12-Week | -2.0 ± 3.2 | -2.5 ± 4.7 | -2.8 ± 2.6 |
| 24-Week / DB 0-Week | -2.3 ± 3.9 | -4.0 ± 6.2 | -3.7 ± 2.8 |
| DB 12-Week |  | -4.8 ± 6.6 | -1.6 ± 3.7 |
| DB 24-Week |  | -3.3 ± 6.6 | -1.7 ± 4.2 |
| DB 36-Week |  | -4.1 ± 6.5 | -1.9 ± 4.0 |
| DB 48-Week |  | -3.0 ± 6.7 | -1.9 ± 4.3 |
