# Supplementary material for: A phase III double-blind, placebo-controlled, randomized withdrawal trial of 5‑aminolevulinic acid hydrochloride with sodium ferrous citrate for efficacy and safety in patients diagnosed as Leigh syndrome
Source: PLoS One. 2026 Jul 17;21(7):e0332283. doi: 10.1371/journal.pone.0332283 (PMC13379092; doi:10.1371/journal.pone.0332283)
Supplement: S4 Table — (DOCX) [file pone.0332283.s004.docx]

**S4 Table.** **Period until discontinuation due to inadequate efficacy of study drug.**

| Group | Patient No | Period until discontinuation from start of the DB-period (Day) |
| --- | --- | --- |
| SPP-004 | ALA-01 | 77 |
| SPP-004 | ALA-12 | 174 |
| Placebo | PLA-01 | 77 |
| Placebo | PLA-04 | 49 |
| Placebo | PLA-07 | 112 |
| Placebo | PLA-08 | 84 |
| Placebo | PLA-11 | 112 |
| Placebo | PLA-12 | 56 |
| Placebo | PLA-13 | 43 |
