# Supplementary material for: A phase III double-blind, placebo-controlled, randomized withdrawal trial of 5‑aminolevulinic acid hydrochloride with sodium ferrous citrate for efficacy and safety in patients diagnosed as Leigh syndrome
Source: PLoS One. 2026 Jul 17;21(7):e0332283. doi: 10.1371/journal.pone.0332283 (PMC13379092; doi:10.1371/journal.pone.0332283)
Supplement: S5 Table — (DOCX) [file pone.0332283.s005.docx]

**S5 Table.** **Changes in cranial nervous symptoms and myopathy symptoms in each patient (baseline to DB-period 48 weeks or at the time of discontinuation)**

| Group | Patient ID | Mobility | Myopathy | Pyramidal | Extra-pyramidal | Ptosis and eye movement | Commu-nication | Hearing | Vision | Selfcare^1)^ | Ataxia^1)^ | Neuro-pathy | Improve  Open period | Long-term  efficacy | Short-term  efficacy | Worsen  DB period | Improve  DB period |
| --- | --- | --- | --- | --- | --- | --- | --- | --- | --- | --- | --- | --- | --- | --- | --- | --- | --- |
| SPP-004 | ALA-09 | ● | ● |  |  | ■ | ● |  |  | ● |  | ● | 5 | 5 | 0 | 0 | 1 |
|  | ALA-07 | ● | ● |  |  |  |  |  |  | ■ | ● |  | 3 | 3 | 0 | 0 | 1 |
|  | ALA-14 |  |  |  |  | ● | ■ | ● | ● |  |  |  | 3 | 3 | 0 | 0 | 1 |
|  | ALA-01 |  |  |  | ○ |  | × |  |  |  |  |  | 2 | 0 | 1 | 1 | 0 |
|  | ALA-08 | ■ | ■ |  |  |  |  | ● | × |  | ■ |  | 2 | 2* | 0 | 1 | 3 |
|  | ALA-13 | ● |  | ● |  |  |  |  |  |  |  |  | 2 | 2 | 0 | 0 | 0 |
|  | ALA-12 |  |  |  |  | × | ○ |  |  |  |  |  | 2 | 0 | 1 | 1 | 0 |
|  | ALA-11 | ● |  |  |  |  |  |  |  |  |  |  | 1 | 1 | 0 | 0 | 0 |
|  | ALA-03 |  |  |  |  | ● |  |  |  |  |  |  | 1 | 1 | 0 | 0 | 0 |
|  | ALA-04 |  |  | ● |  |  | ■ |  |  | - | - |  | 1 | 1 | 0 | 0 | 1 |
|  | ALA-05 |  |  |  |  |  | ● |  |  |  |  |  | 1 | 1 | 0 | 0 | 0 |
|  | ALA-06 |  |  |  |  | ● |  |  |  |  |  |  | 1 | 1 | 0 | 0 | 0 |
|  | ALA-10 | ■ |  |  |  | ● |  |  |  |  |  |  | 1 | 1 | 0 | 0 | 1 |
| Placebo | PLA-08 | × | ○ |  |  |  | ○ |  |  |  |  | ○ | 4 | 0 | 3 | 1 | 0 |
|  | PLA-02 | ● |  |  |  |  |  |  | ● | ● |  |  | 3 | 3 | 0 | 0 | 0 |
|  | PLA-03 | ● | ● | ● |  |  |  |  |  |  |  |  | 3 | 3 | 0 | 0 | 0 |
|  | PLA-13 |  | ○ | × | × |  |  |  |  |  |  |  | 3 | 0 | 1 | 2 | 0 |
|  | PLA-01 | × |  | ○ |  |  |  |  |  |  |  |  | 2 | 0 | 1 | 1 | 0 |
|  | PLA-05 | ● | ● |  |  |  |  |  |  |  |  |  | 2 | 2 | 0 | 0 | 0 |
|  | PLA-07 |  | × | × | × | × |  | ○ |  |  |  |  | 2 | 0 | 1 | 4 | 0 |
|  | PLA-12 |  |  |  | ○ | × |  |  |  |  |  |  | 2 | 0 | 1 | 1 | 0 |
|  | PLA-14 | ● |  | ■ |  |  |  |  |  | - | - |  | 1 | 1 | 0 | 0 | 1 |
|  | PLA-04 |  |  |  |  |  | × |  |  |  |  |  | 1 | 0 | 0 | 1 | 0 |
|  | PLA-06 |  |  |  |  | ● |  |  |  |  |  |  | 1 | 1 | 0 | 0 | 0 |
|  | PLA-09 |  |  |  |  |  |  | ● |  |  |  |  | 1 | 1 | 0 | 0 | 0 |
|  | PLA-10 |  |  |  |  |  |  |  |  |  | ● |  | 1 | 1 | 0 | 0 | 0 |
|  | PLA-11 |  |  |  | × |  |  |  |  |  |  |  | 1 | 0 | 0 | 1 | 0 |
|  |  | Mobility | Myopathy | Pyramidal | Extra-pyramidal | Ptosis and eye movement | Commu-nication | Hearing | Vision | Selfcare^1)^ | Ataxia^1)^ | Neuro-pathy | Total number of items | Total number of patients |  |  |  |
| Open period  efficacy | SPP-004 | 4 | 3 | 2 | 1 | 5 | 4 | 2 | 1 | 1 | 1 | 1 | 25 | 13 |  |  |  |
|  | Placebo | 6 | 5 | 3 | 3 | 2 | 2 | 2 | 1 | 1 | 1 | 1 | 27 | 14 |  |  |  |
| Long-term  efficacy | SPP-004 | 4 | 4^2)^ | 2 | 0 | 4 | 2 | 2 | 1 | 1 | 1 | 1 | 21 | 11 |  |  |  |
|  | Placebo | 4 | 2 | 1 | 0 | 1 | 0 | 1 | 1 | 1 | 1 | 0 | 12 | 7 |  |  |  |
| Short-term  efficacy | SPP-004 | 0 | 0 | 0 | 0 | 0 | 0 | 0 | 0 | 0 | 0 | 0 | 2 | 2 |  |  |  |
|  | Placebo | 0 | 0 | 0 | 0 | 0 | 0 | 0 | 0 | 0 | 0 | 0 | 7 | 5 |  |  |  |
| Worsen  DB period | SPP-004 | 0 | 0 | 0 | 0 | 1 | 1 | 0 | 1 | 0 | 0 | 0 | 3 | 3 |  |  |  |
|  | Placebo | 2 | 1 | 2 | 3 | 2 | 1 | 0 | 0 | 0 | 0 | 0 | 11 | 7 |  |  |  |
| Improve  DB period | SPP-004 | 2 | 1 | 0 | 0 | 1 | 2 | 0 | 0 | 1 | 1 | 0 | 8 | 6 |  |  |  |
|  | Placebo | 0 | 0 | 1 | 0 | 0 | 0 | 0 | 0 | 0 | 0 | 0 | 1 | 1 |  |  |  |

Open period: Open-label period

Improvement during Open-label period, ●: Long-term efficacy: Improvement in the open-label period was maintained until the end of the study (≥44 weeks in the DB-period), ○: Short-term efficacy: Improvement in the open-label period was maintained until the end of the study (<44 weeks in the DB-period), ■: Improvement in the DB-period, ×: worsen in the DB-period. 1): Self-care and Ataxia are specific-items for 2 years-old and older
