# Supplementary material for: A phase III double-blind, placebo-controlled, randomized withdrawal trial of 5‑aminolevulinic acid hydrochloride with sodium ferrous citrate for efficacy and safety in patients diagnosed as Leigh syndrome
Source: PLoS One. 2026 Jul 17;21(7):e0332283. doi: 10.1371/journal.pone.0332283 (PMC13379092; doi:10.1371/journal.pone.0332283)
Supplement: S6 Table — (DOCX) [file pone.0332283.s006.docx]

**S6 Table.** **Summary of efficacy of each item of cranial nervous symptoms and myopathy symptoms in the DB-period.**

| item | FAS-DB period (SPP-004: *n* =13, Placebo: *n* =14) | | | |
| --- | --- | --- | --- | --- |
|  | Maintainance^1)^ or Improvement | | inadequate efficacy of study drug | |
|  | SPP-004 | Placebo | SPP-004 | Placebo |
| Mobility | 4/4 (100%) | 4/6 (66.7%) | 0/4 (0.0%) | 2/6 (33.3%) |
| Myopathy | 3/3 (100%) | 4/5 (80.0%) | 0/3 (0.0%) | 1/5 (20.0%) |
| Pyramidal | 2/2 (100%) | 2/3 (66.7%) | 0/2 (0.0%) | 1/3 (33.3%) |
| Extrapyramidal | 1/1 (100%) | 1/3 (33.3%) | 0/1 (0.0%) | 2/3 (66.7%) |
| Ptosis and Eye movement | 4/5 (80.0%) | 1/2 (50.0%) | 1/5 (20.0%) | 1/2 (50.0%) |
| Communication | 3/4 (75.0%) | 1/2 (50.0%) | 1/4 (25.0%) | 1/2 (50.0%) |
| Hearing | 2/2 (100%) | 2/2 (100%) | 0/2 (0.0%) | 0/2 (0.0%) |
| Vision | 1/1 (100%) | 1/1 (100%) | 0/1 (0.0%) | 0/1 (0.0%) |
| Selfcare^2)^ | 1/1 (100%) | 1/1 (100%) | 0/1 (0.0%) | 0/1 (0.0%) |
| Ataxia^2)^ | 1/1 (100%) | 1/1 (100%) | 0/1 (0.0%) | 0/1 (0.0%) |
| Neuropathy | 1/1 (100%) | 1/1 (100%) | 0/1 (0.0%) | 0/1 (0.0%) |

FAS-DB period: FAS in the double-blind period

The ratio (percentage) of maintenance or improvement and worsening for symptomatic patients (Score >0) for the item at baseline are indicated.

1) Both of short-term and long-term efficacy maintenance are included.

2) Selfcare and Ataxia are specific-items for 2 years-old and older
