# Supplementary material for: A phase III double-blind, placebo-controlled, randomized withdrawal trial of 5‑aminolevulinic acid hydrochloride with sodium ferrous citrate for efficacy and safety in patients diagnosed as Leigh syndrome
Source: PLoS One. 2026 Jul 17;21(7):e0332283. doi: 10.1371/journal.pone.0332283 (PMC13379092; doi:10.1371/journal.pone.0332283)
Supplement: S7 Table — The improvement ratio indicates the percentage of the number of improved items to the number of symptomatic items at baseline. (DOCX) [file pone.0332283.s007.docx]

**S7 Table.** **Summary of changes in NPMDS scores for** **cranial nervous symptoms and myopathy symptoms in notably improved patients (ALA-08 and ALA-09). The improvement ratio indicates the percentage of the number of improved items to the number of symptomatic items at baseline.**

| Patient ID | Group | Improvement | | | Worsening |
| --- | --- | --- | --- | --- | --- |
|  |  | Open-label period | DB-period | Overall period | DB-period |
| ALA-08 | SPP-004 | Improvement ratio: 33.3% (2/6)  Myopathy: Severe to Moderate  Hearing: Moderate to Mild | Improvement ratio:50.0% (3/6)  Myopathy: Moderate to Normal  Ataxia: Severe to Moderate  Mobility: Severe to Moderate | Improvement ratio:66.7% (4/6)  Myopathy: Severe to Normal  Hearing: Moderate to Mild  Ataxia: Severe to Moderate  Mobility: Severe to Moderate | Vision: Normal to Mild |
| ALA-09 | SPP-004 | Improvement ratio:50.0% (5/10)  Mobility: Severe to Moderate  Myopathy: Moderate to Mild  Communication: Moderate to Mild  Selfcare: Severe to Moderate  Myopathy: Severe to Moderate | Improvement ratio:10.0% (1/10)  Ptosis and Eye movement: Mild to Normal | Improvement ratio:60.0%（6/10）  Mobility: Severe to Moderate  Myopathy: Moderate to Mild  Communication: Moderate to Mild  Selfcare: Severe to Moderate  Myopathy: Severe to Moderate  Ptosis and Eye movement: Mild to Normal | None |
