# Supplementary material for: A phase III double-blind, placebo-controlled, randomized withdrawal trial of 5‑aminolevulinic acid hydrochloride with sodium ferrous citrate for efficacy and safety in patients diagnosed as Leigh syndrome
Source: PLoS One. 2026 Jul 17;21(7):e0332283. doi: 10.1371/journal.pone.0332283 (PMC13379092; doi:10.1371/journal.pone.0332283)
Supplement: S8 Table — (DOCX) [file pone.0332283.s008.docx]

**S8 Table.** **Summary of adverse events (overall study period*)**

|  | SPP-004 | |
| --- | --- | --- |
|  | *n* =54 | |
|  | Cases (%) | Events |
| All adverse events | 49 (90.7) | 239 |
| Severity |  |  |
| Mild | 25 (46.3) | 165 |
| Moderate | 20 (37.0) | 69 |
| Severe | 4 (7.4) | 5 |
| Causal relationship with study drugs |  |  |
| Unrelated | 42 (77.8) | 232 |
| Related | 7 (13.0) | 7 |
| Death | 0 (0.0) | - |
| Serious adverse events | 16 (29.6) | 40 |
| Adverse events leading to discontinuation of study drug | 1 (1.9) | 1 |

* Open-label period+ SPP-004 group in the DB-period.
