# Supplementary material for: A phase III double-blind, placebo-controlled, randomized withdrawal trial of 5‑aminolevulinic acid hydrochloride with sodium ferrous citrate for efficacy and safety in patients diagnosed as Leigh syndrome
Source: PLoS One. 2026 Jul 17;21(7):e0332283. doi: 10.1371/journal.pone.0332283 (PMC13379092; doi:10.1371/journal.pone.0332283)
Supplement: S9 Table — (DOCX) [file pone.0332283.s009.docx]

**S9 Table.** **Adverse drug reaction for the open-label period and the DB-period (SAS)**

| **SOC** | | **Open-label period (0-24 weeks)** |  | **DB-period (0-48 weeks)** | | |
| --- | --- | --- | --- | --- | --- | --- |
|  |  | **All (*n* = 54)** |  | **SPP-004 (*n* =14)** |  | **Placebo (*n* =14)** |
|  | **PT** | **Cases (%)** |  | **Cases (%)** |  | **Cases (%)** |
| **Psychiatric disorders** | | **1 (1.9)** |  | - |  |  |
|  | Insomnia | 1 (1.9) | - | - |  | - |
| **Respiratory, thoracic, and mediastinal disorders** | | **1 (1.9)** |  | - | - |  |
|  | Cough | 1 (1.9) | - | - |  | - |
| **Gastrointestinal disorders** | | **5 (9.3)** |  | - | - |  |
|  | Tooth discolouration | 4 (7.4) | - | - |  | - |
|  | Vomiting | 1 (1.9) | - | - |  | - |
| **Metabolism and nutrition disorders** | | - |  | **0 (0.0)** |  | **1 (7.1)** |
|  | Diabetes mellitus | - |  | 0 (0.0) |  | 1 (7.1) |
| **Overall** | | **7 (13.0)** |  | **0 (0.0)** |  | **1 (7.1)** |

SOC: System Organ Class

PT: Preferred Term

MedDRA/J Ver. 23.0
