# Supplementary material for: A phase III double-blind, placebo-controlled, randomized withdrawal trial of 5‑aminolevulinic acid hydrochloride with sodium ferrous citrate for efficacy and safety in patients diagnosed as Leigh syndrome
Source: PLoS One. 2026 Jul 17;21(7):e0332283. doi: 10.1371/journal.pone.0332283 (PMC13379092; doi:10.1371/journal.pone.0332283)
Supplement: S10 Table — (DOCX) [file pone.0332283.s010.docx]

**S10 Table.** **Serious adverse events (overall study period*)**

| **SOC** | **PT** | **n (%)** | **Number of Incidence** | **Patient** |
| --- | --- | --- | --- | --- |
| **Infections and infestations** | | **12 (22.2)** | **18** |  |
|  | Pneumonia | 5 (9.3) | 6 | OD-03, OD-13, ALA-10, ALA-13, PLA-06 |
|  | Bronchitis | 3 (5.6) | 3 | OD-03, OD-13, ALA-10 |
|  | Bacteraemia | 1 (1.9) | 1 | ALA-11 |
|  | Gastroenteritis | 1 (1.9) | 1 | OD-13 |
|  | Gastroenteritis adenovirus | 1 (1.9) | 1 | ALA-14 |
|  | Gastroenteritis viral | 1 (1.9) | 1 | ALA-07 |
|  | Influenza | 1 (1.9) | 1 | ALA-07 |
|  | Nasopharyngitis | 1 (1.9) | 1 | OD-20 |
|  | Respiratory syncytial virus bronchiolitis | 1 (1.9) | 1 | ALA-14 |
|  | Respiratory syncytial virus infection | 1 (1.9) | 1 | OD-12 |
|  | Gastroenteritis norovirus | 1 (1.9) | 1 | ALA-13 |
| **Metabolism and nutrition disorders** | | **1 (1.9)** | **1** |  |
|  | Dehydration | 1 (1.9) | 1 | ALA-14 |
| **Nervous system disorders** | | **4 (7.4)** | **8** |  |
|  | Seizure | 1 (1.9) | 5 | ALA-10 |
|  | Epilepsy | 1 (1.9) | 1 | OD-26 |
|  | Myoclonus | 1 (1.9) | 1 | ALA-03 |
|  | Status epilepticus | 1 (1.9) | 1 | OD-13 |
| **Respiratory, thoracic and mediastinal disorders** | | **4 (7.4)** | **10** |  |
|  | Upper respiratory tract inflammation | 3 (5.6) | 3 | OD-15, OD-26, ALA-14 |
|  | Increased upper airway secretion | 1 (1.9) | 3 | ALA-14 |
|  | Pleural effusion | 1 (1.9) | 2 | ALA-06 |
|  | Pneumonia aspiration | 1 (1.9) | 2 | ALA-14 |
| **Gastrointestinal disorders** | | **2 (3.7)** | **2** |  |
|  | Vomiting | 1 (1.9) | 1 | OD-26 |
|  | Oesophageal rupture | 1 (1.9) | 1 | ALA-02 |
| **General disorders and administration site conditions** | | **1 (1.9)** | **1** |  |
|  | Pyrexia | 1 (1.9) | 1 | ALA-13 |
| **Overall** |  | **16 (29.6)** | **40** |  |

*: Open-label period+ SPP-004 group in the DB-period.

SOC: System Organ Class, PT: Preferred Term, MedDRA/J Ver. 23.0, OD: open-label-discontinued group, ALA: SPP-004 group, PLA: Placebo group
