# Supplementary figures and images for: A phase III double-blind, placebo-controlled, randomized withdrawal trial of 5‑aminolevulinic acid hydrochloride with sodium ferrous citrate for efficacy and safety in patients diagnosed as Leigh syndrome
Source: PLoS One. 2026 Jul 17;21(7):e0332283. doi: 10.1371/journal.pone.0332283 (PMC13379092; doi:10.1371/journal.pone.0332283)

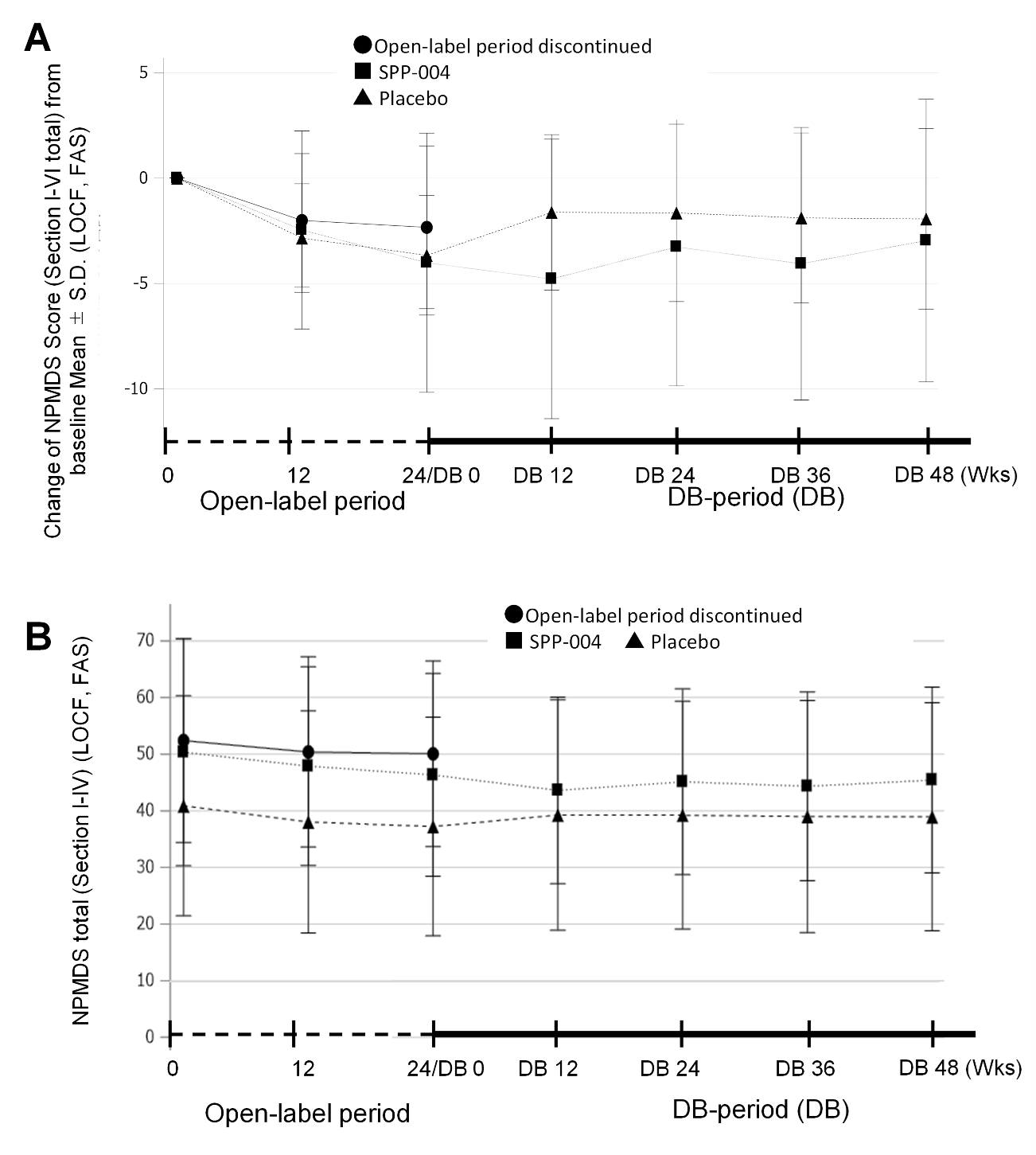

Supplement: S1 Fig — (TIF) [file pone.0332283.s011.tif]

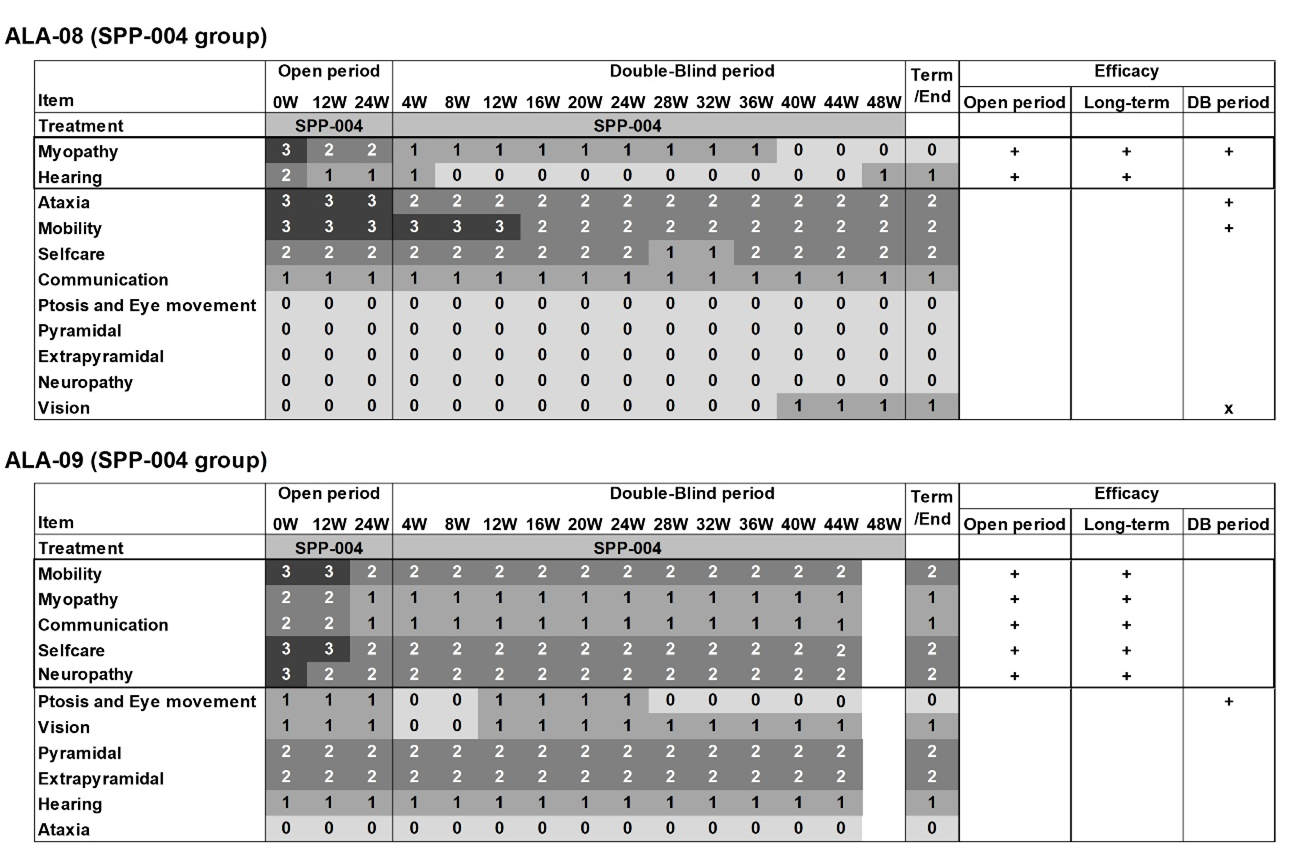

Supplement: S3 Fig — (TIF) [file pone.0332283.s013.tif]

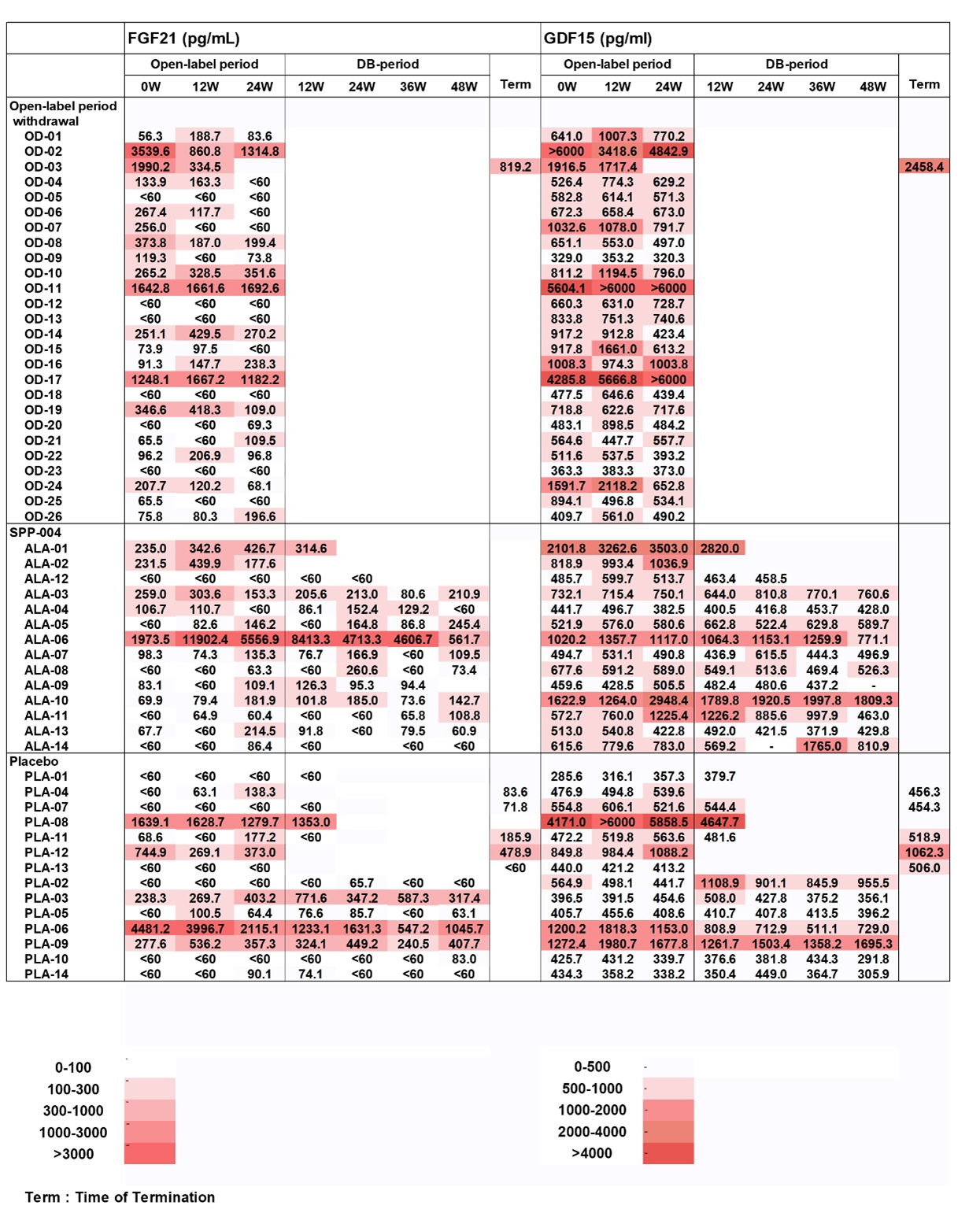

Supplement: S4 Fig — (TIF) [file pone.0332283.s014.tif]

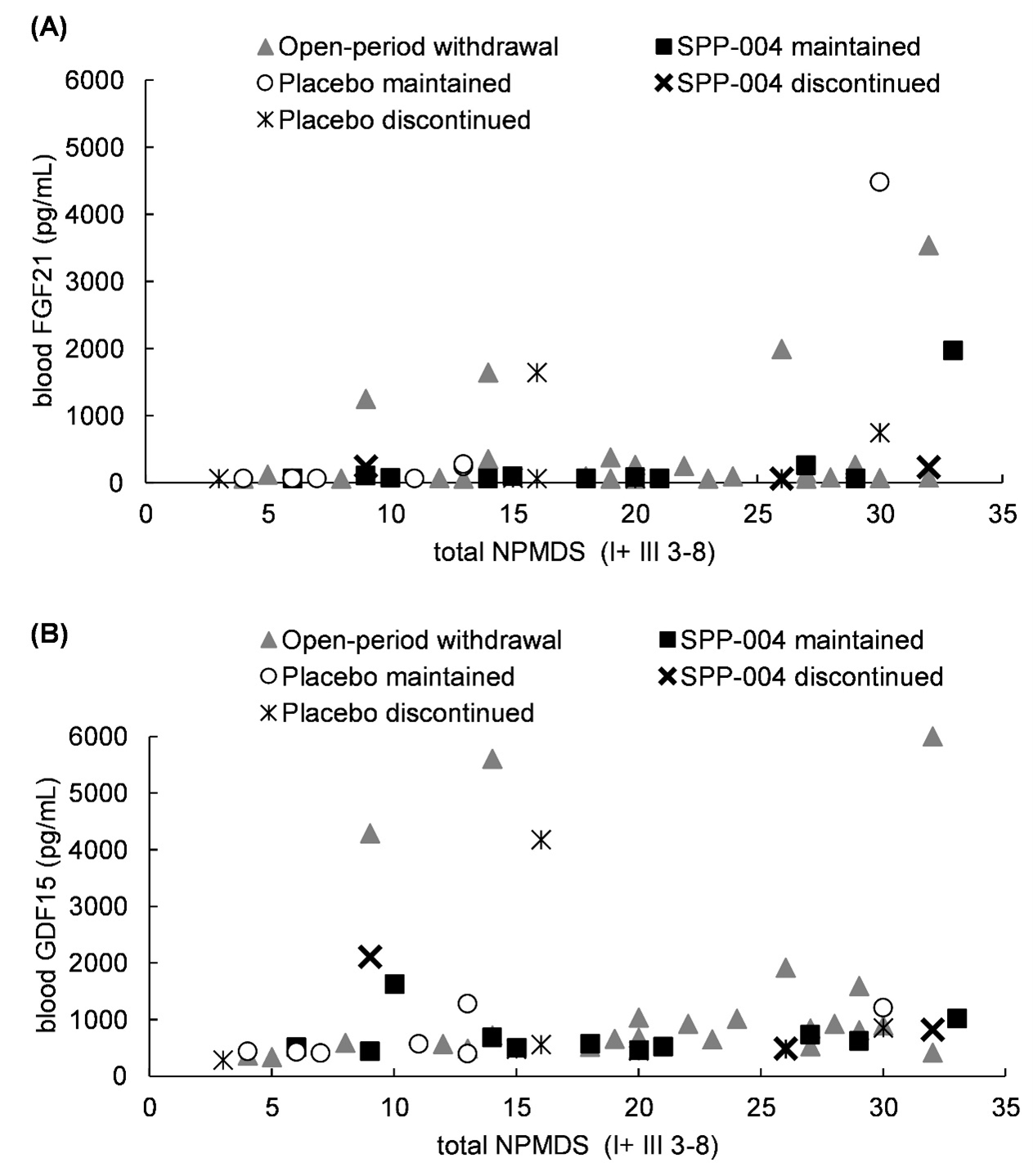

Supplement: S5 Fig — (TIF) [file pone.0332283.s015.tif]

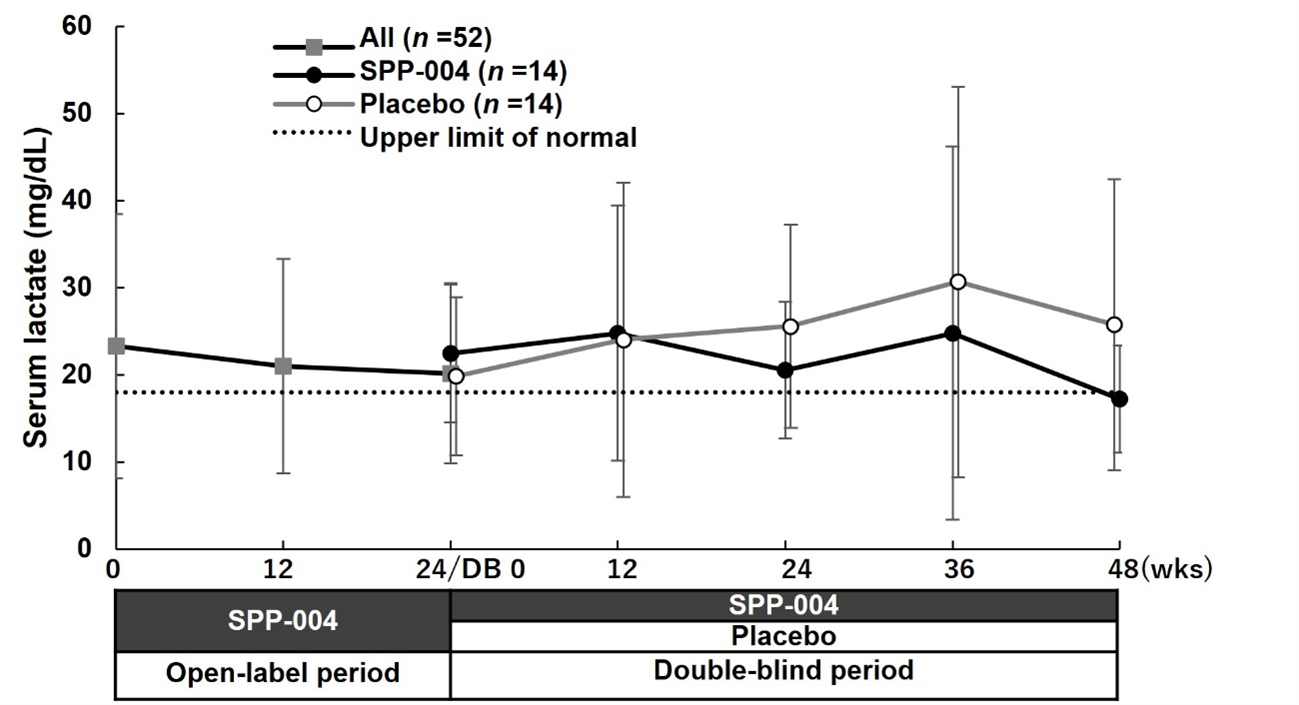

Supplement: S6 Fig — (TIF) [file pone.0332283.s016.tif]
