# Supplementary material for: A phase III double-blind, placebo-controlled, randomized withdrawal trial of 5‑aminolevulinic acid hydrochloride with sodium ferrous citrate for efficacy and safety in patients diagnosed as Leigh syndrome
Source: PLoS One. 2026 Jul 17;21(7):e0332283. doi: 10.1371/journal.pone.0332283 (PMC13379092; doi:10.1371/journal.pone.0332283)
Supplement: S2 Fig — (PDF) [file pone.0332283.s012.pdf]

S2 Figure.

Mobility

Group	Patient	Open period			Double-Blind period												Term	Efficacy		
Group	Patient	0W	12W	24W	4W	8W	12W	16W	20W	24W	28W	32W	36W	40W	44W	48W	/End	Open period	Long-term	DB period
SPP-004	Treatment	SPP-004			SPP-004															
	ALA-07	3	3	2	2	2	2	2	2	2	2	2	2	2	2	2	2	+	+	
	ALA-11	3	3	2	2	2	2	2	2	2	2	2	2	2	2	2	2	+	+	
	ALA-09	3	3	2	2	2	2	2	2	2	2	2	2	2	2		2	+	+	
	ALA-13	2	2	1	1	1	1	1	1	1	1	1	1	1	1	1	1	+	+	
	ALA-08	3	3	3	3	3	3	2	2	2	2	2	2	2	2	2	2			+
	ALA-10	1	3	2	2	2	1	1	1	1	1	1	1	1	1	1	1	-		+
Placebo	Treatment	SPP-004			Placebo															
	PLA-03	3	2	2	2	2	2	2	2	2	2	2	2	2	2	2	2	+	+	
	PLA-02	3	3	2	2	2	2	2	2	2	2	2	2	2	2	2	2	+	+	
	PLA-05	2	1	1	1	1	1	1	1	1	1	1	1	1	1	1	1	+	+	
	PLA-14	2	2	1	1	1	1	1	1	1	1	1	1	1	1	1	1	+	+	
	PLA-08	3	2	2	2	3	3										3	+		-
	PLA-01	1	1	0	0	1	1										1	+		-

Myopathy

Group	Patient	Open period			Double-Blind period												Term	Efficacy		
Group	Patient	0W	12W	24W	4W	8W	12W	16W	20W	24W	28W	32W	36W	40W	44W	48W	/End	Open period	Long-term	DB period
SPP-004	Treatment	SPP-004			SPP-004															
	ALA-08	3	2	2	1	1	1	1	1	1	1	1	1	0	0	0	0	+	+	+
	ALA-07	3	3	1	1	2	1	1	1	1	1	1	1	1	1	1	1	+	+	
	ALA-09	2	2	1	1	1	1	1	1	1	1	1	1	1	1		1	+	+	
	ALA-10	1	1	2	2	2	2	2	2	2	2	2	2	2	2	2	2	-		
Placebo	Treatment	SPP-004			Placebo															
	PLA-03	2	1	1	1	1	1	1	1	1	1	1	1	1	1	1	1	+	+	
	PLA-05	2	1	1	1	1	1	1	1	1	1	1	1	1	1	1	1	+	+	
	PLA-07	3	2	2	2	2	3	3									3	+		-
	PLA-08	3	2	2	2	2	2										2	+		
	PLA-13	2	1	1	1	1											1	+		

Pyramidal

Group	Patient	Open period			Double-Blind period												Term	Efficacy		
Group	Patient	0W	12W	24W	4W	8W	12W	16W	20W	24W	28W	32W	36W	40W	44W	48W	/End	Open period	Long-term	DB period
SPP-004	Treatment	SPP-004			SPP-004															
	ALA-13	2	2	1	1	1	1	1	1	1	1	1	1	1	1	1	1	+	+	
	ALA-04	1	0	0	0	0	0	0	0	0	0	0	0	0	0	0	0	+	+	
Placebo	Treatment	SPP-004			Placebo															
	PLA-03	2	1	1	1	1	1	1	1	1	1	1	1	1	1	1	1	+	+	
	PLA-01	1	1	0	0	0	0										0	+		
	PLA-13	3	2	2	3	3											3	+		-
	PLA-14	1	1	1	1	1	1	1	1	1	1	1	0	0	0	0	0			+
	PLA-07	0	0	0	0	0	2	2									2			-

Extra pyramidal

Group	Patient	Open period 0W	12W	24W	Double-Blind period 4W	8W	12W	16W	20W	24W	28W	32W	36W	40W	44W	48W	Term /End	Efficacy Open period	Long-term	DB period
SPP-004	Treatment	SPP-004			SPP-004															
	ALA-01	2	2	1	1	1	1										1	+		
	ALA-10	0	0	1	1	1	1	1	1	1	1	1	1	1	1	1	1	-		
Placebo	Treatment	SPP-004			Placebo															
	PLA-12	3	3	2	2	2											2	+		
	PLA-11	3	3	2	3	2	3	3									3	+		-
	PLA-13	2	1	1	2	2											2	+		-
	PLA-07	2	2	2	3	2	3	3									3			-

Ptosis and Eyemovement

Group	Patient	Open period 0W	12W	24W	Double-Blind period 4W	8W	12W	16W	20W	24W	28W	32W	36W	40W	44W	48W	Term /End	Efficacy Open period	Long-term	DB period
SPP-004	Treatment	SPP-004			SPP-004															
	ALA-10	3	3	2	2	2	1	1	1	1	1	1	1	1	1	1	1	+	+	+
	ALA-03	3	2	2	2	2	2	2	2	2	2	2	2	2	2	2	2	+	+	
	ALA-06	3	3	2	2	2	2	2	2	2	2	2	2	2	2	2	2	+	+	
	ALA-14	1	1	0	0	1	0	0	0	-	0	0	0	0	0	0	0	+	+	
	ALA-12	3	2	2	2	2	2	2	3	3							3	+		-
	ALA-09	1	1	1	0	0	1	1	1	1	0	0	0	0	0		0			+
	ALA-04	1	1	1	1	1	1	1	0	1	1	1	1	1	1	1	1			
Placebo	Treatment	SPP-004			Placebo															
	PLA-06	3	2	2	2	2	2	2	2	2	2	2	2	2	2	2	2	+	+	
	PLA-12	3	3	2	3	3											3	+		-
	PLA-07	0	0	0	0	0	2	2									2			-

Communication

Group	Patient	Open period 0W	12W	24W	Double-Blind period 4W	8W	12W	16W	20W	24W	28W	32W	36W	40W	44W	48W	Term /End	Efficacy Open period	Long-term	DB period
SPP-004	Treatment	SPP-004			SPP-004															
	ALA-05	2	1	1	1	1	1	1	1	1	1	1	1	1	1	1	1	+	+	
	ALA-09	2	2	1	1	1	1	1	1	1	1	1	1	1	1		1	+	+	
	ALA-12	2	1	1	1	1	1	2	1	1							1	+		
	ALA-01	2	2	1	1	2	2										2	+		-
	ALA-14	3	3	3	3	2	2	2	2	-	2	2	2	2	2	2	2			+
	ALA-04	2	2	2	2	2	2	2	2	2	2	2	2	2	2	1	1			+
Placebo	Treatment	SPP-004			Placebo															
	PLA-08	1	0	0	0	0	0										0	+		
	PLA-04	2	2	1	2	2											2	+		-

Hearing

Group	Patient	Open period			Double-Blind period												Term	Efficacy		
		0W	12W	24W	4W	8W	12W	16W	20W	24W	28W	32W	36W	40W	44W	48W	/End	Open period	Long-term	DB period
SPP-004	Treatment	SPP-004			SPP-004															
	ALA-14	3	3	2	2	1	1	1	1	-	1	1	1	1	1	1	1	+	+	+
	ALA-08	2	1	1	1	0	0	0	0	0	0	0	0	0	0	1	1	+	+	
Placebo	Treatment	SPP-004			Placebo															
	PLA-09	2	2	1	1	1	1	1	1	1	1	1	1	1	1	1	1	+	+	
	PLA-07	1	0	0	0	0	0	0									0	+		

Vision

Group	Patient	Open period			Double-Blind period												Term	Efficacy		
		0W	12W	24W	4W	8W	12W	16W	20W	24W	28W	32W	36W	40W	44W	48W	/End	Open period	Long-term	DB period
SPP-004	Treatment	SPP-004			SPP-004															
	ALA-14	2	2	1	1	2	1	1	1	-	1	1	1	1	1	1	1	+	+	
	ALA-09	1	1	1	0	0	1	1	1	1	1	1	1	1	1		1			
	ALA-08	0	0	0	0	0	0	0	0	0	0	0	0	1	1	1	1			-
Placebo	Treatment	SPP-004			Placebo															
	PLA-02	2	2	1	1	1	1	1	1	1	1	1	1	1	1	1	1	+	+	

Selfcare

Group	Patient	Open period			Double-Blind period												Term	Efficacy		
		0W	12W	24W	4W	8W	12W	16W	20W	24W	28W	32W	36W	40W	44W	48W	/End	Open period	Long-term	DB period
SPP-004	Treatment	SPP-004			SPP-004															
	ALA-09	3	3	2	2	2	2	2	2	2	2	2	2	2	2		2	+	+	
	ALA-07	3	3	3	3	3	3	3	3	3	3	3	2	2	2	2	2			+
	ALA-08	2	2	2	2	2	2	2	2	2	1	1	2	2	2	2	2			
Placebo	Treatment	SPP-004			Placebo															
	PLA-02	2	2	1	1	1	1	1	1	1	1	1	1	1	1	1	1	+	+	

Ataxia

Group	Patient	Open period			Double-Blind period												Term	Efficacy		
		0W	12W	24W	4W	8W	12W	16W	20W	24W	28W	32W	36W	40W	44W	48W	/End	Open period	Long-term	DB period
SPP-004	Treatment	SPP-004			SPP-004															
	ALA-07	3	3	2	2	2	2	2	2	2	2	2	2	2	2	2	2	+	+	
	ALA-08	3	3	3	2	2	2	2	2	2	2	2	2	2	2	2	2			+
	ALA-10	1	1	2	2	2	2	2	2	2	2	2	2	2	2	2	2	-		
Placebo	Treatment	SPP-004			Placebo															
	PLA-10	2	2	1	1	1	1	1	1	1	1	1	1	1	1	1	1	+	+	

Neuropathy

Group	Patient	Open period			Double-Blind period												Term	Efficacy		
		0W	12W	24W	4W	8W	12W	16W	20W	24W	28W	32W	36W	40W	44W	48W	/End	Open period	Long-term	DB period
SPP-004	Treatment	SPP-004			SPP-004															
	ALA-09	3	2	2	2	2	2	2	2	2	2	2	2	2	2		2	+	+	
	ALA-14	2	2	2	2	3	2	2	2	-	2	2	2	2	2	2	2			
Placebo	Treatment	SPP-004			Placebo															
	PLA-08	3	2	2	2	2	2										2	+		
