# Supplementary material for: A phase III double-blind, placebo-controlled, randomized withdrawal trial of 5‑aminolevulinic acid hydrochloride with sodium ferrous citrate for efficacy and safety in patients diagnosed as Leigh syndrome
Source: PLoS One. 2026 Jul 17;21(7):e0332283. doi: 10.1371/journal.pone.0332283 (PMC13379092; doi:10.1371/journal.pone.0332283)
Supplement: Attachment — Submitted filename: report_10.1101+2025.08.31.25334800.pdf [file pone.0332283.s020.pdf]

ScreenIT Report

This is an automated report for 10.1101+2025.08.31.25334800.pdf. This report was solicited by the PLOS One editorial team and provided by ScreenIT.

ScreenIT is an independent group of scientists developing automated tools that analyze academic papers. A set of automated tools screened your submitted manuscript and provided the report below. Each tool was created by your academic colleagues with the goal of helping authors.

The tools look for factors that are important for transparency, rigor and reproducibility, and we hope that the report might help you to improve reporting in your manuscript. Within the report you will find links to more information about the items that the tools check. These links include helpful papers, websites, or videos that explain why the item is important.

While our screening tools aim to improve and maintain quality standards they may, on occasion, miss nuances specific to your study type or flag something incorrectly. Each tool has limitations that are described on the ScreenIT website. The tools screen the main file for the paper; they are not able to screen supplements stored in separate files.

Please note that the Academic Editor had access to these comments while making a decision on your manuscript. The Academic Editor may ask that issues flagged in this report be addressed.

If you would like to provide feedback on the ScreenIT tool, please email the team at ScreenIt@bih-charite.de. If you have questions or concerns about the review process, please contact the PLOS One office at plosone@plos.org.

How to read the report:

- no action required
- review for potential issues
- a critical issue was found by a tool
- may not be applicable to your paper

- more information about this item can be obtained using this link

Rigor

✓	Ethics	IRB: "The protocol of this phase III study was reviewed and approved by the institutional review board of each study site as well as the relevant regulatory agency, and it was conducted in compliance with good clinical practice (GCP)."<br>Consent: "Patients who can stop taking foods containing 5-ALA-phosphate 28 days before the start of administration (if they are taking 5-ALA-phosphate-containing foods at the time of informed consent). 4."
✓	Inclusion and Exclusion Criteria	"Patients Japanese patients with LS who were mainly involved in central nervous system disorders consented to participate and were judged to be eligible."
✓	Flow charts and attrition	Thank you for including a study flow chart and information about attrition and exclusions to help readers evaluate the risk of bias.<br><br>Sentence about attrition: "Similar analyses were also planned to be performed for prognosis regarding death as an event but were not performed because no patient was dead in this study."
✓	Sex as a biological variable	"Patients who participated in other clinical trials within 12 weeks before obtaining consent. 7. Pregnant women, lactating women or patients who may be pregnant. 8."
✓	Subject Demographics	Age: "The patient (or legal representative) consented to enter the DB-period *: Items of NPMDS for cranial nervous symptoms and myopathy symptoms Section I: Hearing, Communication, self-care, mobility Section III: vision, ptosis and eye movement, myopathy, ataxia, pyramidal, extrapyramidal, neuropathy. (Self-care and ataxia are specific items for people 2 years old and older.) Criteria for inadequate efficacy of the study drug in the DB-period 1."
✓	Randomization	"multicenter, randomized withdrawal, placebo-controlled, double-blind, parallel-group, confirmatory study (SPED-ALA-003

		study) was conducted from July 2018 to May 2020 at eight institutions, including Saitama Medical School Hospital, Hokkaido University Hospital, Jichi Medical University Hospital"
✓	Blinding	"DB-SAS: Safety analysis set (SAS) in the double blind (DB)-period, DB-FAS: Full analysis set (FAS) in the DB-period, DB-PPS: Per protocol set (PPS) in the DB-period Because LS is a severe disease with poor prognosis, conducting a traditional placebocontrolled, double-blind study may cause ethical concerns that patients in the placebo group remain untreated for a long period."
?	Power Analysis	Not detected. Please state how you determined the sample size for the study. If you performed a power or sample size calculation, please provide details.
n/a	Replication	not required.

Generated by Barzooka (flow charts), SciScore (everything else).
SciScore only screens the text of the methods section; we apologize if the tool has missed items reported elsewhere in the paper or supplements.

Resources

Persistent unique identifiers for research resources, RRIDs, can be used to track these key resources though the literature. They are requested or required in many journals, but even if your journal does not require them, we strongly encourage the use of these for the readers of papers who wish to easily track down the key materials like antibodies, software tools, or mice.

Software and Algorithms	"Blood FGF21 was measured by using ELISA kits for FGF21 (BioVendor, Brno, Czech Republic) in Dr."	? BioVendor suggested: BioVendor Laboratory Medicine (RRID:SCR_005143). Please make sure this RRID suggestion is accurate before including in your manuscript.

Generated by SciScore.
SciScore only screens the text of the methods section; we apologize if the tool has missed items reported elsewhere in the paper or supplements.

Transparency

?	Open code	If you wrote code to analyze your data, please consider sharing the code in a public repository. This makes it easier for others to reproduce your analyses, and may also aid others seeking to analyze similar datasets.
?	Open data	If permitted, sharing data on a public repository with a persistent identifier (a DOI or accession number) can improve reproducibility and make it easier for other scientists to expand on your work. Papers with open data are cited more often than papers without open data. Some institutions have an expert who can provide advice on data sharing.
✓	Self-acknowledged limitations	"39 / 59 The limitation of this confirmation trial was partly due to the unavailability of information on LS-associated genetic alterations identified in each patient. Over 75 genes in mitochondrial and nuclear DNA have been identified as a cause of LS, and the symptoms and prognosis depend on the abnormality of the causal genes [33,34]. Shimura et al. [15] reported that skin fibroblasts harvested from six out of eight patients with MD including LS responded to 5-ALA treatment and improved oxidative phosphorylation and increased ATP biosynthesis in mitochondria. In contrast, two of them did not show any response to 5-ALA. Considering those facts, we may speculate a relationship between the causal gene abnormality and the severity of LS, as well as the response to SPP-004. Additional investigation of the gene alterations in each patient with LS may help to identify patients who could benefit from SPP-004 treatment."
✓	Conflict of interest statement	Thank you for stating any conflicts of interest.
✓	Funding statement	Thank you for reporting your funding source(s), or stating that there was no specific funding for this work.
n/a	Registration statement	A registered protocol makes the planned research project more transparent and can protect against bias. Please consider registering future confirmatory studies.

Generated by ODDPub, limitation-recognizer, rtransparent, trial-identifier.

Figures

✓	No bar graphs of continuous data	ℹ Thank you for not using bar graphs to present continuous data. Many different datasets and data distributions can lead to the same bar graph, and the actual data may suggest different conclusions from the summary statistics alone.
✓	No rainbow color maps	ℹ Thank you for not using rainbow colormaps. Rainbow color maps distort readers’ perception of the data by introducing visual artifacts and are not accessible to colorblind readers.

Generated by JetFighter, Barzooka.
