# Supplementary material for: A phase III double-blind, placebo-controlled, randomized withdrawal trial of 5‑aminolevulinic acid hydrochloride with sodium ferrous citrate for efficacy and safety in patients diagnosed as Leigh syndrome
Source: PLoS One. 2026 Jul 17;21(7):e0332283. doi: 10.1371/journal.pone.0332283 (PMC13379092; doi:10.1371/journal.pone.0332283)
Supplement: Attachment — Submitted filename: PLOS_ONE_LS_Ran_Comments.docx [file pone.0332283.s019.docx]

**Subject:** Review of Manuscript – PONE-D-25-46225 - [EMID:9175fe2d96d786e0]

Dear Editor and Authors,

Thank you for the opportunity to review this manuscript reporting the results of a Phase 3 trial in LS. Conducting a clinical trial in a rare pediatric disease such as LS is an extremely challenging and admirable undertaking. I commend the authors for their significant efforts in designing, executing, and reporting this important study.

Overall, the manuscript is well written and provides valuable data for the LS clinical and research community. The study appears to have been well executed, and the reporting is generally clear and comprehensive. However, I have several concerns regarding the analysis and interpretation of the results that I believe need to be addressed to strengthen the manuscript. My detailed comments are as follows:

**Major Comments**

1. **Patient Discontinuations During the Open-Label (OL) Period (Lines 285–291)**
   According to the manuscript, 26 patients discontinued during the OL period. This represents a relatively high dropout rate. Based on the described study criteria, it appears these patients did not experience any clinical improvement during the OL phase.
   - Could the authors provide more detail on the reasons for these discontinuations?
   - If approximately half of the patients did not improve during the OL period, it raises questions about the interpretation of the positive results seen in the double-blind (DB) period. A deeper discussion of this point would be very helpful to readers in understanding the true clinical benefit.
2. **Patient Withdrawal from the SPP and Inclusion in Analysis Sets**
   The manuscript notes that one patient withdrew from the SPP.
   - Please clarify the reason for this withdrawal and why this patient was excluded from the Full Analysis Set (FAS).
   - Given the small sample size of the study, it is critical to be cautious when excluding patients, especially from the FAS, as this could introduce bias and affect the robustness of the results.
   - I strongly recommend performing a **sensitivity analysis** that includes this patient to evaluate whether the study’s findings remain consistent.
   - Since this patient was included in the Safety Analysis Set (SAS), inclusion in the efficacy analysis would provide a more complete picture of the study results and improve transparency.
3. **Clarity of Figure 3 Legend**
   In Figure 3, the placebo group includes a symbol labeled “Mb.” However, this symbol is not defined in the figure legend. Please clarify what “Mb” represents to ensure the figure is fully interpretable.
4. **High Rate of Upper Respiratory Events and Infections (SAEs)**
   The manuscript reports a relatively high frequency of upper respiratory events and infections categorized as Serious Adverse Events (SAEs).
   - Could the authors provide potential explanations for these findings?
   - It would be helpful to know whether these events were considered related to the study drug, underlying disease, or external factors (e.g., seasonal illness).
   - A brief discussion on how these safety signals compare to similar studies in this patient population would provide valuable context for clinicians and researchers.
5. **Context of Current Standard Clinical Interventions (Introduction, Line 110)**
   In the introduction, the authors state that there are currently no approved drugs for LS. While this is important to note, it would also be helpful to provide a brief overview of **current standard non-pharmacological clinical interventions** or management strategies used to alleviate symptoms. This additional context would help readers better understand the clinical landscape and unmet medical need that this trial addresses.

**General Comments**

- While the study design and execution are commendable, certain aspects of the **analysis and interpretation** lack clarity. Addressing the points above will significantly improve the transparency and robustness of the manuscript.
- Given the small sample size and challenges inherent in rare disease trials, it is especially important to ensure that all decisions regarding patient inclusion/exclusion and data interpretation are fully justified and clearly communicated.
- I encourage the authors to include additional details in the Discussion to help readers better understand the implications of the study’s findings for both clinical practice and future research.

With revisions to address the concerns noted above, I believe this manuscript has the potential to make a strong contribution to the literature.

I would be pleased to review a revised version once these issues have been addressed.

Thank you again for the opportunity to review this important work.

Sincerely,
Ran Liao, PhD
Eli Lilly and Company
