# Supplementary material for: A phase III double-blind, placebo-controlled, randomized withdrawal trial of 5‑aminolevulinic acid hydrochloride with sodium ferrous citrate for efficacy and safety in patients diagnosed as Leigh syndrome
Source: PLoS One. 2026 Jul 17;21(7):e0332283. doi: 10.1371/journal.pone.0332283 (PMC13379092; doi:10.1371/journal.pone.0332283)
Supplement: Attachment — Submitted filename: Response to Reviewers_PONE-D-25-46225.doc [file pone.0332283.s022.doc]

December 8, 2025

To the Editorial Team at *PLOS One*:

We are grateful to you for your evaluation and consideration of our Original Article for publication in *PLOS One,* entitled “**A phase III double-blind, placebo-controlled, randomized withdrawal trial of 5-aminolevulinic acid hydrochloride with sodium ferrous citrate for efficacy and safety in patients diagnosed as** **Leigh syndrome**.”

According to the valuable advice and suggestions provided by the reviewers and the editor, we completed our revision of the manuscript and herein submit the revised manuscript with our replies to Reviewers’ comments point by point.

Regarding the COI, SBI Pharmaceuticals supported this clinical study by providing research funds and salaries to its own employees only.

We hope the revised manuscript is now acceptable for publication in PLOS ONE

Sincerely,

Motowo Nakajima, Ph.D.

Director, SBI Pharmaceuticals Co., Ltd.,

Izumi Garden Tower 13F

1-6-1 Roppongi, Minato-ku, Tokyo 106-6013, Japan

Phone: +81-3-6229-0095

Fax: +81-3-3589-0761

Email: motnakaj@sbigroup.co.jp

Website: https://sbipharma.co.jp/english/

**Reply to Reviewer 1**

Subject**:** Review of Manuscript – PONE-D-25-46225 - [EMID:9175fe2d96d786e0]

Dear Editor and Authors,

Thank you for the opportunity to review this manuscript reporting the results of a Phase 3 trial in LS. Conducting a clinical trial in a rare pediatric disease such as LS is an extremely challenging and admirable undertaking. I commend the authors for their significant efforts in designing, executing, and reporting this important study.

Overall, the manuscript is well written and provides valuable data for the LS clinical and research community. The study appears to have been well executed, and the reporting is generally clear and comprehensive. However, I have several concerns regarding the analysis and interpretation of the results that I believe need to be addressed to strengthen the manuscript. My detailed comments are as follows:

**Major Comments**

1. **Patient Discontinuations During the Open-Label (OL) Period (Lines 285–291)**
   According to the manuscript, 26 patients discontinued during the OL period. This represents a relatively high dropout rate. Based on the described study criteria, it appears these patients did not experience any clinical improvement during the OL phase.
   - Could the authors provide more detail on the reasons for these discontinuations?

The reason for withdrawal during the OL period was a lack of improvement in the NPMDS scores for cranial nervous symptoms and myopathy symptoms. To clarify the reasons for withdrawal, which were not previously stated, we have added this information on lines 290-292.

We distinguish between failure to transition from the OL period to the DB period as “withdrawal” and discontinuation during the DB period as “discontinuation”. In Table 1, Figure 1 and Figure 3, the terms were used incorrectly, so we have corrected them.

- If approximately half of the patients did not improve during the OL period, it raises questions about the interpretation of the positive results seen in the double-blind (DB) period. A deeper discussion of this point would be very helpful to readers in understanding the true clinical benefit.

As the reviewer pointed out, approximately half of the patients did not show improvement with SPP-004 administration during the OL period. However, as explained in the introduction, LS are genetically and biologically heterologous. Symptoms vary widely, and severity differs from patient to patient. Considering these factors, it is not surprising that approximately half of the cases did not show improvement during OL period. This idea is also supported by the fact that in vitro study using fibroblasts from MD patients by Shimura et al. (2019), improvement in ATP production or ORR was dependent on the gene mutations.

On the other hand, the efficacy in the DB phase represents a maintenance of the improvement achieved by SPP-004, that is, the effect maintained in patients for whom SPP-004 was effective during OL period. Therefore, we believe that the dropout of half of the patients during the OL period does not call into question the efficacy of SPP-004 in the DB period.

We have added a discussion on the dependence of the efficacy of SPP-004 on genetic mutations and symptoms in lines 562-571.

2. **Patient Withdrawal from the SPP and Inclusion in Analysis Sets**
   The manuscript notes that one patient withdrew from the SPP.
   - Please clarify the reason for this withdrawal and why this patient was excluded from the Full Analysis Set (FAS).

In this case, the SAE occurred immediately after the DB stage, so efficacy evaluation during the DB stage could never be performed. This has been added to the main text.

- Given the small sample size of the study, it is critical to be cautious when excluding patients, especially from the FAS, as this could introduce bias and affect the robustness of the results.

The comment of the reviewer was reasonable. However, we believe that the exclusion of the case was not arbitrary and does not pose any bias that would affect the robustness of the results.

- I strongly recommend performing a **sensitivity analysis** that includes this patient to evaluate whether the study’s findings remain consistent.

As mentioned above, there is no efficacy data for the DB period in this case, so it is not possible to include this in the efficacy analysis.

- Since this patient was included in the Safety Analysis Set (SAS), inclusion in the efficacy analysis would provide a more complete picture of the study results and improve transparency.

As mentioned above, there is no efficacy data for this case in the DB period, so it is considered appropriate to exclude it from the efficacy analysis. The validity of the study has been added to the revised manuscript, and the transparency of the research results is clear.

3. **Clarity of Figure 3 Legend**
   In Figure 3, the placebo group includes a symbol labeled “Mb.” However, this symbol is not defined in the figure legend. Please clarify what “Mb” represents to ensure the figure is fully interpretable.

Thank you for pointing this out. The symbol and legend were inconsistent, so we have unified them both to Mb.

4. **High Rate of Upper Respiratory Events and Infections (SAEs)**
   The manuscript reports a relatively high frequency of upper respiratory events and infections categorized as Serious Adverse Events (SAEs).
   - Could the authors provide potential explanations for these findings?

We added the explanations of these SAEs in the discussions (Lines 574-

582).

- It would be helpful to know whether these events were considered related to the study drug, underlying disease, or external factors (e.g., seasonal illness).

We added the descriptions in the discussions. They are unrelated with the study drug and most events were due to accidental infections.

- A brief discussion on how these safety signals compare to similar studies in this patient population would provide valuable context for clinicians and researchers.

We added a brief discussion by citing a report by Sofou et al. (2014) that describes disease courses and predictors of survival in patients with LS.

5. **Context of Current Standard Clinical Interventions (Introduction, Line 110)**
   In the introduction, the authors state that there are currently no approved drugs for LS. While this is important to note, it would also be helpful to provide a brief overview of **current standard non-pharmacological clinical interventions** or management strategies used to alleviate symptoms. This additional context would help readers better understand the clinical landscape and unmet medical need that this trial addresses.

In response to this comment, we have provided more details about clinical interventions and approved medications for LS (Lines 89-95 in the revised manuscript).

**Reply to Reviewer 2: Screen IT Report**

**Rigor**

**Power analysis: Not detected**

We explained the sample size limitation due to our difficulties in recruitment of very rare disease LS patients in Japan only. Further investigations with enough numbers of LS patients in different countries and areas are necessary for any power analysis.

**Resources**

**Software and Algorithms: BioVendor suggested: BioVendor Laboratory Medicine (RRID:SCR_005143)**

We could not confirm BioVenodr’s suggestion at their home page [Immunoassays | BioVendor Research and Diagnostics Products](https://www.biovendor.com/immunoassays). Therefore, we kept the product name in the methods instead of adding the suggested catalogue number (RRID:SCR_005143).

**Transparency**

**Open Code and Open Data: If permitted, sharing data on a public open repository.**

As mentioned in the manuscript, the clinical data and records are owned by each of the participants of the study and those are available on an appropriate official request from academic institutions for further investigation.

**Reply to Reviewer 3**

**1. Methods:
Methods reporting need some work. An orderly manner is suggested, following CONSORT guidelines, without repeating information, such as Trial Design, Participant Eligibility Criteria and settings, Interventions, Outcomes, sample size/power considerations, Interim analysis and stopping rules, Randomization (details on random number generation, allocation concealment, implementation), Blinding issues, etc, should be mentioned. The authors are advised to create "separate subsections" for each of the possible topics (whichever necessary), and that way produce a very clear writeup. They are advised to write it carefully, following nice examples in the manuscript below:**

[**https://www.sciencedirect.com/science/article/pii/S0889540619300010**](https://www.sciencedirect.com/science/article/pii/S0889540619300010)

**We have revised the description in accordance with the Consort guidelines.**

**Specific comments:
(a) For instance, the randomization and allocation concealment should be made very clear (they are NOT the same thing); the trial staff recruiting patients should NOT have the randomization list. Randomization should be prepared by the trial statistician, and he/she would not participate in the recruiting.**

We clearly described randomization and allocation concealment (lines 177-181). The randomization key code is managed by the Allocation manager, who is not involved in patient recruitment.

**(b) More details on the randomization is needed; I could find no such details. Was it a block randomization? What block size?**

We used the permuted block randomization method. Block size was not described because it was not disclosed. **(c) Sample size: The sample size/power statement should mention the "name of the specific test used", and the corresponding effect size desired. Also, a separate paragraph would serve the readers better.**

We describe the sample size and effective size on lines 182-189.

**(d) Statistical Analysis writeup

(d1) The statistical analysis writeup proposes LOCFs to impute missing values (from early discontinuation). That the LOCF method has significant limitations is already noted in the literature. Is it possible for the authors to assess the sensitivity of this LOCF-based imputation, to something like multiple imputation technique, often implemented via the MICE algorithm; see here:** [**https://www.jstatsoft.org/article/view/v045i03**](https://www.jstatsoft.org/article/view/v045i03)

We understand the limitations of the LOCF method. However, if NPMDS scores are not adjusted, discrepancies may occur, such as an improvement in the mean score due to the dropout of patients whose symptoms worsened.

Adjustment using the Mice algorithm was not performed, as we were unaware of this method at the time of planning this study.

**(d2) The statistical methods presented are restricted to only finding confidence interval for the ratio, using Clopper-Pearson method. I believe, during the trial, various subject-level covariates were also collected. Was there any particular reason, why a subsequent regression approach was not considered, and statistical analysis only restricted to finding ratios and their confidence intervals?**

As you pointed out, various subject-level covariates were also collected during the trial. However, the study had a small sample size of 15 cases in each group at the time of planning, which was deemed insufficient for regression analysis. Therefore, we did not perform regression analysis.

**(d3) Kaplan-Meier methods were used (Figure 2A), but those were never mentioned in the statistical analysis writeup.**

We described Kaplan-Meier analysis in lines 225-228. Statistical comparison was performed by log-rank test as described in lines 229-230.

**2. Results & Conclusions:

(a) The authors should check that any statement of significance should be followed by a p-value in the entire Results section. Otherwise, the Results section look OK; it's pretty straightforward.**

We confirmed that p-values were reported for items tested for significance. **(b) Conclusions should state that the current findings are** **ONLY based on the samples derived from the Japanese population and should allude to future studies with much larger sample sizes and collected at other geographical areas to confirm the effectiveness of the SPP-004.**

In response to the comments, the conclusion section now includes a discussion of future studies
