# Supplementary material for: A phase III double-blind, placebo-controlled, randomized withdrawal trial of 5‑aminolevulinic acid hydrochloride with sodium ferrous citrate for efficacy and safety in patients diagnosed as Leigh syndrome
Source: PLoS One. 2026 Jul 17;21(7):e0332283. doi: 10.1371/journal.pone.0332283 (PMC13379092; doi:10.1371/journal.pone.0332283)
Supplement: Attachment — Submitted filename: Responces to reviewers_PONE-D-25-46225-R2.docx [file pone.0332283.s023.docx]

May 12, 2026

Dr. Bhanwar Lal Puniya, Ph.D.
Academic Editor
PLOS One

Re:　PONE-D-25-46225R1

A phase III double-blind, placebo-controlled, randomized withdrawal trial of 5‑aminolevulinic acid hydrochloride with sodium ferrous citrate for efficacy and safety in patients diagnosed as Leigh syndrome

Dear Dr. Bhanwar Lal Puniya,

Thank you very much for providing your kind advice and comments for improving our first revision of PONE-D-25-46225R1. We added further information to clarify our findings and made changes to our descriptions according to the suggestions provided by you and the reviewers. We explained those changes and improvements in the second revision point by point in our reply as follows. Those changes made in the second revised manuscript were highlighted. We hope our answers and further information added to the second revision are sufficient to clarify all your questions and concerns.

We would appreciate it if you could review our second revision in a timely manner and consider its acceptance for publication in PLOS One.

Sincerely yours,

Motowo Nakajima, Ph.D.

Director, SBI Pharmaceuticals Co., Ltd.

Executive Director, NX Development Corporation, Lexington, KY

Visiting Professor, Yamagata University School of Medicine

Visiting Professor, Keio University Graduate School of Health Management

Izumi Garden Tower 13F, 1-6-1 Roppongi

Minato-ku, Tokyo 106-6013, JAPAN

Tel: +81-3-6229-0095

E-mail: [motnakaj@sbigroup.co.jp](mailto:motnakaj@sbigroup.co.jp)

<https://sbipharma.co.jp/english/>

Dear Dr. Nakajima,

Thank you for submitting your manuscript to PLOS ONE. After careful consideration, we feel that it has merit but does not fully meet PLOS ONE’s publication criteria as it currently stands. Therefore, we invite you to submit a revised version of the manuscript that addresses the points raised during the review process.

=======================================
The manuscript has clearly benefited from the revisions made in response to the previous reviewers. The manuscript has now been evaluated by an additional reviewer, whose comments are enclosed and should be addressed in detail, in addition to those from the previous reviewers.  In addition to those points, I have also listed my concerns that need further clarification before the manuscript can be considered for publication.

=======================================

Please submit your revised manuscript by May 30 2026 11:59PM. If you will need more time than this to complete your revisions, please reply to this message or contact the journal office at [plosone@plos.org](mailto:plosone@plos.org). When you're ready to submit your revision, log on to <https://www.editorialmanager.com/pone/> and select the 'Submissions Needing Revision' folder to locate your manuscript file.

Please include the following items when submitting your revised manuscript:

- A letter that responds to each point raised by the academic editor and reviewer(s). You should upload this letter as a separate file labeled 'Response to Reviewers'.
- A marked-up copy of your manuscript that highlights changes made to the original version. You should upload this as a separate file labeled 'Revised Manuscript with Track Changes'.
- An unmarked version of your revised paper without tracked changes. You should upload this as a separate file labeled 'Manuscript'.

If you would like to make changes to your financial disclosure, please include your updated statement in your cover letter. Guidelines for resubmitting your figure files are available below the reviewer comments at the end of this letter.

If applicable, we recommend that you deposit your laboratory protocols in protocols.io to enhance the reproducibility of your results. Protocols.io assigns your protocol its own identifier (DOI) so that it can be cited independently in the future. For instructions see: [https://journals.plos.org/plosone/s/submission-guidelines#loc-laboratory-protocols](https://track.editorialmanager.com/CL0/https:%2F%2Fjournals.plos.org%2Fplosone%2Fs%2Fsubmission-guidelines%23loc-laboratory-protocols/1/010f019d945bb446-6969edb1-867a-4136-b18a-9166b80428f6-000000/y_Ny9fexKDhAPd1d8UHcgyDrwspo1WdH8tNA9ThPRQQ=258). Additionally, PLOS ONE offers an option for publishing peer-reviewed Lab Protocol articles, which describe protocols hosted on protocols.io. Read more information on sharing protocols at [https://plos.org/protocols?utm_medium=editorial-email&utm_source=authorletters&utm_campaign=protocols](https://track.editorialmanager.com/CL0/https:%2F%2Fplos.org%2Fprotocols%3Futm_medium=editorial-email%26utm_source=authorletters%26utm_campaign=protocols/1/010f019d945bb446-6969edb1-867a-4136-b18a-9166b80428f6-000000/lw6iwUBXwIjbps0afziGQBad-uaf6NGSd0ELDuuSyq8=258).

As the corresponding author, your ORCID iD is verified in the submission system and will appear in the published article. PLOS supports the use of ORCID, and we encourage all coauthors to register for an ORCID iD and use it as well. Please encourage your coauthors to verify their ORCID iD within the submission system before final acceptance, as unverified ORCID iDs will not appear in the published article. *Only* the individual author can complete the verification step; PLOS staff *cannot* verify ORCID iDs on behalf of authors.

We look forward to receiving your revised manuscript.

Kind regards,

Bhanwar Lal Puniya, Ph.D.
Academic Editor
PLOS One

Journal Requirements:

1. If the reviewer comments include a recommendation to cite specific previously published works, please review and evaluate these publications to determine whether they are relevant and should be cited. There is no requirement to cite these works unless the editor has indicated otherwise.

   Additional Editor Comments:

   (1) In some sections, it is indicated that no specific funding was received. At the same time, in the cover letter it is stated that SBI Pharmaceuticals supported the study and provided salaries to some authors. Please revise the funding and competing interest sections, and clearly describe the role of the sponsor, if any, in study design, data collection, analysis, and manuscript preparation.

>>Answer to Editor: According to the editor’s advice the role of the sponsor, SBI Pharmaceuticals, was clearly described in the declaration of Competing Interest. The roles of the employees of SBI Pharmaceuticals were shown in the co-authors’ contributions.

(2) Please check the data availability statements. At one point it is stated that all data are fully available, while elsewhere it is suggested that data are only available upon request. Please provide a clear data availability statement that is accurate and complies with journal policy.　>> Answer to Editor: Thank you for your advice. We made our descriptions of data availability consistent throughout the manuscript. All the data is fully available (lines 723-725).

(3) There needs to be a clearer presentation is the interpretation of the primary endpoint. As reported, the primary endpoint does not reach statistical significance, while the abstract and conclusions place more emphasis on positive findings from secondary analyses. The authors are encouraged to present a more balanced interpretation. It would help readers if the manuscript clearly states that the primary endpoint was not statistically significant and frames the other findings as supportive/exploratory, rather than definitive.

>> Answer to Editor: We carefully checked our conclusions in Abstract and Discussion and clearly stated that the primary endpoint was not statistically significant and the other findings were framed as supportive/exploratory, rather than definitive.

(4) Because only patients who improved during the open-label phase entered the randomized phase, the double-blind portion of the study evaluates maintenance of response in a selected group of responders.
This is a reasonable approach. However, it limits the generalizability of the findings. Please state this more explicitly (in the abstract and discussion), so that the scope of the conclusions is clear.
>> Answer to Editor:

We thank the reviewer for this constructive comment. We agree that, because only patients who showed clinical improvement during the open‑label phase were randomized, the double‑blind phase evaluates maintenance of response in a responder‑enriched population, which limits generalizability.

To address this, we have revised the Abstract (lines 46-53, lines 61-62) to explicitly state that the study objective and conclusions concern maintenance of therapeutic benefit in patients who achieved an initial response. We have also revised the Discussion to clearly acknowledge this design feature as a limitation and to note that the findings should be interpreted within this context (line 589-600).

(5) The differences between groups in sex distribution and age at onset are noticeable because of small sample size. While this may be unavoidable, it would be helpful to acknowledge this more clearly as a limitation and add a comment on its possible impact on interpretation.
>> Answer to Editor: Although the differences between groups in sex distribution and age at onset were noticeable, there is a limitation of its possible impact on interpretation due to small sample size. This limitation has now been more clearly stated in the Discussion (lines 659-668), together with an explanation that recruitment of a larger and more balanced cohort was challenging because Leigh syndrome is a rare disease.

(6) The use of the LOCF method and the exclusion of one randomized patient from the efficacy analysis would benefit from additional clarification. In light of the additional reviewer’s comments, it would also be helpful to clarify how missing data and follow-up were handled, particularly regarding the optional Week-48 follow-up and the transition to the extension phase.
A brief clarification of (i) the rationale for using LOCF, (ii) its limitations, and (iii) how the analysis population was defined would improve transparency.
>> Answer to Editor:

We thank the reviewer for this helpful comment. We have revised the Statistical analysis section to clarify the rationale for using the last‑observation‑carried‑forward (LOCF) method, the handling of missing data and follow‑up, and the definition of the analysis population. Specifically, we now explain that LOCF was used to handle missing efficacy data due to early discontinuation and to support consistent longitudinal comparisons (lines 280-283). We also clarify that when the Week‑48 follow‑up was not completed because of early discontinuation or transition to the extension study, efficacy was evaluated using the last available assessment obtained at discontinuation or prior to transition (lines 284–286).

Furthermore, we have already described that the double‑blind efficacy analysis population (FAS) included all randomized patients with at least one post‑randomization efficacy assessment, and that one randomized patient was excluded due to the absence of any post‑randomization efficacy data following an early serious adverse event (lines 299-301). A brief statement acknowledging the limitations of the LOCF approach has also been added to the Discussion (lines 666–668).

(7) A careful pass for language and clarity would help improve readability.
>> Answer to Editor: The additional editorial work was performed by a native English scientist to improve readability.
[Note: HTML markup is below. Please do not edit.]

Reviewer's Responses to Questions

**Comments to the Author**

1. If the authors have adequately addressed your comments raised in a previous round of review and you feel that this manuscript is now acceptable for publication, you may indicate that here to bypass the “Comments to the Author” section, enter your conflict of interest statement in the “Confidential to Editor” section, and submit your "Accept" recommendation.

Reviewer #1: All comments have been addressed

Reviewer #3: All comments have been addressed

Reviewer #4: (No Response)

2. Is the manuscript technically sound, and do the data support the conclusions?

The manuscript must describe a technically sound piece of scientific research with data that supports the conclusions. Experiments must have been conducted rigorously, with appropriate controls, replication, and sample sizes. The conclusions must be drawn appropriately based on the data presented.

Reviewer #1: Yes

Reviewer #3: (No Response)

Reviewer #4: (No Response)

3. Has the statistical analysis been performed appropriately and rigorously?

Reviewer #1: Yes

Reviewer #3: (No Response)

Reviewer #4: (No Response)

4. Have the authors made all data underlying the findings in their manuscript fully available?

The [PLOS Data policy](http://track.editorialmanager.com/CL0/http:%2F%2Fwww.plosone.org%2Fstatic%2Fpolicies.action%23sharing/1/010f019d945bb446-6969edb1-867a-4136-b18a-9166b80428f6-000000/peY-4MdTEfvz1YYbUn3cGVeLLANyPh-4VL5uS7-lo6w=258) requires authors to make all data underlying the findings described in their manuscript fully available without restriction, with rare exception (please refer to the Data Availability Statement in the manuscript PDF file). The data should be provided as part of the manuscript or its supporting information, or deposited to a public repository. For example, in addition to summary statistics, the data points behind means, medians and variance measures should be available. If there are restrictions on publicly sharing data—e.g. participant privacy or use of data from a third party—those must be specified.

Reviewer #1: No

Reviewer #3: (No Response)

Reviewer #4: (No Response)

5. Is the manuscript presented in an intelligible fashion and written in standard English?

PLOS ONE does not copyedit accepted manuscripts, so the language in submitted articles must be clear, correct, and unambiguous. Any typographical or grammatical errors should be corrected at revision, so please note any specific errors here.

Reviewer #1: Yes

Reviewer #3: Yes

Reviewer #4: (No Response)

6. Review Comments to the Author

Please use the space provided to explain your answers to the questions above. You may also include additional comments for the author, including concerns about dual publication, research ethics, or publication ethics. (Please upload your review as an attachment if it exceeds 20,000 characters)

Reviewer #1: Thank you to the author team for amending the manuscript and addressing my comments - I appreciate the effort. The manuscript has indeed improved considerably. I don't have further comments on the data itself, but I do have a concern about whether the author team has disclosed all data underlying the findings and made it fully available. I did not find any listing of patients with SAEs or patient narratives; the manuscript currently contains only summary-level tables and figures for the description of the data. I am fine with this approach if it aligns with PLOS data policy, but I wanted to point this out for clarification.

Answer to Reviewer #2:

In the table of SAE (S10 table) in the open label and double-blind periods, not only the number of patients but also their IDs were included in each SAE case. A few patients showed more than 2 SAEs, however, these were often related to infections. We included these data and observations in the results.

Reviewer #3: (No Response)

Reviewer #4: Summary of the Manuscript
The authors report a multicenter, double-blind, placebo-controlled, randomized withdrawal trial of an investigational agent targeting mitochondrial dysfunction in 54 patients aged 7.9 ± 6.52 with Leigh syndrome. The study included a 24-week open-label period followed by a 48-week double-blind period. The study was conducted between July 2018 and May 2020. The primary endpoint was the change from baseline in the Newcastle Pediatric Mitochondrial Disease Scale (NPMDS) score, section I and III, at Week 48. The primary efficacy endpoint was the proportion of patients discontinuing due to inadequate efficacy by Week 48. Secondary endpoints included the proportions of patients who discontinued due to inadequate efficacy at Weeks 24 and 36.

Major Strengths
This rare-disease trial in a challenging population is addressing a critical unmet need in mitochondrial medicine. I congratulate the authors for successfully combining an open-label lead-in with a randomized, double-blind, placebo-controlled phase, logistically difficult in progressive neurodegenerative disorders, while also monitoring both clinical and biomarker endpoints. Use of the NPMDS shows commitment to a disease-specific efficacy measure, and high retention during the open-label period shows strong operational execution. The manuscript is well-structured and thoughtfully incorporates multiple angles.

Questions, Concerns, and Suggestions
1. The protocol amendment made Week-48 follow-up optional for patients entering the long-term extension. Patients perceiving benefits are more likely to skip formal follow-up and enter extension early, leading to LOCF carrying forward last positive observations. This might inflate apparent efficacy. Please report the number of people who exercised the optional early-entry pathway and skipped mandatory follow-up.
>> Answer to Reviewer #4:
We thank the reviewer for raising this important point regarding the potential impact of the protocol amendment.

All patients who entered the long‑term extension without completing the　48-week DB-period (**n=9**) discontinued the double‑blind study due to insufficient efficacy; no early entry was driven by perceived clinical benefit. Thus, the concern that favorable outcomes were preferentially carried forward by LOCF is not supported by the data. This has been explained in the result section (lines 304-306).

In addition, the amendment making the follow‑up (at 48-week or at time of discontinuation) optional was implemented **from a** patient‑protection perspective, with the intention of minimizing the treatment‑free period after discontinuation for insufficient efficacy, rather than to selectively advance patients with favorable responses.

2. The Exploratory trial that has been referred to (Abe et al., 2025) mentions “NPMDS section II and section III items 1 and 2 were not suitable for the evaluation of the results of these studies, as their substantial observation periods (3, 4, and 6 months) were longer than that of the study period (12 weeks for both the double- blind and open-label periods)”. However, the current study has a period of 24 weeks followed by 48 weeks (6 months and 12 months respectively).
Citing references/reasoning, if any, that support the modified subscale would be helpful. I strongly recommend reporting the full NPMDS total score as a key secondary endpoint, if available.
>> Answer to Reviewer #4:

We thank the reviewer for the important suggestion regarding inclusion of full NPMDS data.

In response, we have added the **full** NPMDS longitudinal data as Supplemental Figure 1. As expected, the full NPMDS demonstrated greater inter‑individual variability than the modified NPMDS for cranial nervous symptoms and myopathy symptoms (Lines 380-391), reflecting the heterogeneous and slowly progressive nature of Leigh syndrome.

Despite this increased variability, a trend toward maintenance of improvement was more frequently observed in the SPP‑004 group, whereas placebo‑treated patients showed greater fluctuation and progression over the 48‑week double‑blind period. These findings are consistent with the primary analysis and support the interpretation that SPP‑004 may contribute to stabilization rather than short‑term symptomatic change.

We have also clarified in the Discussion that, due to the limited sensitivity of several full NPMDS domains over short‑term follow‑up, these results should be interpreted cautiously and primarily as supportive evidence (lines 589-600).

3. In untreated Leigh syndrome, natural history data show ~+2 points worsening on the full NPMDS over ~48 weeks (Lim et al. 2021). In your findings, on the modified subscale, placebo patients worsened on average from −1.9 ± 1.00 to −1.1 ± 1.83 (mean change +0.8), yet the large SD (±1.83) indicates that some individuals still showed improvement even on placebo. The SPP-004 group demonstrated a trend toward improvement (−1.6 ± 1.82 to −2.3 ± 2.18; mean −0.7).
Given the tiny between-group difference (~1.5 points) and high variability, pls comment on the robustness of the findings with respect to expected short-term variability of a slowly progressive condition. Pls consider adding full NPMDS data to add value.
Are there any clear differences between the placebo and SPP-004 groups baselines at the start of 48 weeks?
>> Answer to Reviewer #4:

We thank the reviewer for highlighting the issue of baseline comparability.

As shown in Supplemental Table 3, patients in the SPP-004 group had higher full NPMDS scores at the start of the double-blind period (mean ± SD: 50.3 ± 20.1) compared with the placebo group (40.9 ± 19.4), indicating a greater baseline disease severity in the SPP-004 group.

This imbalance suggests that patients randomized to SPP-004 were, on average, more severely affected at baseline. It was a factor that would be expected to bias outcomes toward less improvement rather than favoring an apparent treatment effect. Despite this disadvantage, the SPP-004 group demonstrated overall maintenance or improvement in both the modified NPMDS subscale and the full NPMDS score during the double-blind period, whereas the placebo group showed a tendency toward stabilization or mild worsening.

Given the progressive nature of Leigh syndrome and the approximately +2-point deterioration over 48 weeks reported in untreated patients in natural history studies (Lim et al., 2021), the observed trajectories suggest that SPP-004 may have attenuated disease progression even in a more severely affected population.

We acknowledge that the small sample size, baseline imbalance, and substantial inter-individual variability limit definitive conclusions. Accordingly, these findings should be interpreted as supportive rather than confirmatory evidence of treatment benefit. We have revised the Results (lines 380-391) and Discussion sections (lines 589-600) to explicitly address this baseline difference and its implications for robustness and interpretation.

4. An imbalance in SAEs was observed during the double-blind period (64.3% [9/14] in the SPP-004 group vs 7.1% [1/14] in placebo). The authors attribute the high incidence of upper respiratory tract infections to incidental infections or underlying Leigh syndrome, and state they are not causally related to the study drug. However, if these events are due to incidental infections or disease progression, the incidence should have been comparable between arms, especially since the placebo group showed disease worsening. Clarity on how causality was formally assessed and whether any SAEs were considered “possibly” or “probably” related to the drug would be helpful.

>> Answer to Reviewer #4:
We thank the reviewer for this important comment regarding the numerical imbalance in serious adverse events (SAEs) observed during the double-blind period.

All SAEs were formally assessed for causality by the investigators in accordance with the study protocol and Good Clinical Practice criteria. As a result of this systematic assessment, none of the reported SAEs, including those occurring during the double-blind period, was considered possibly or probably related to SPP-004.

To improve clarity, we have revised the Results section to explicitly state that all SAEs were judged as not related to the study drug. In addition, Table 5 has been updated to include a specific category for serious adverse drug reactions (serious adverse reactions), clearly demonstrating that no serious adverse reactions were observed in either treatment group.

Furthermore, we have revised the Discussion section to acknowledge the numerical imbalance in SAE incidence between treatment arms and to clarify that this imbalance should be interpreted with caution, given the small sample size, baseline heterogeneity, and the well-recognized susceptibility of patients with Leigh syndrome to intercurrent infections independent of study treatment.

These clarifications have been added to better address the reviewer’s concern and to strengthen the interpretation of the safety findings.

7. PLOS authors have the option to publish the peer review history of their article ([what does this mean?](https://track.editorialmanager.com/CL0/https:%2F%2Fjournals.plos.org%2Fplosone%2Fs%2Feditorial-and-peer-review-process%23loc-peer-review-history/1/010f019d945bb446-6969edb1-867a-4136-b18a-9166b80428f6-000000/yr0jepDRzsOULqKO4w3OnTklLjhdfcabbIsPcarmegc=258)). If published, this will include your full peer review and any attached files.

If you choose “no”, your identity will remain anonymous but your review may still be made public.

**Do you want your identity to be public for this peer review?** For information about this choice, including consent withdrawal, please see our [Privacy Policy](https://track.editorialmanager.com/CL0/https:%2F%2Fwww.plos.org%2Fprivacy-policy/1/010f019d945bb446-6969edb1-867a-4136-b18a-9166b80428f6-000000/v6Cl0s9pfJdfC89smvCaUJcMtyO23mad26Qo84-KsfE=258).

Reviewer #1: **Yes:** Ran Liao

Reviewer #3: No

Reviewer #4: No

[NOTE: If reviewer comments were submitted as an attachment file, they will be attached to this email and accessible via the submission site. Please log into your account, locate the manuscript record, and check for the action link "View Attachments". If this link does not appear, there are no attachment files.]
To ensure your figures meet our technical requirements, please review our figure guidelines: <https://journals.plos.org/plosone/s/figures>

You may also use PLOS’s free figure tool, NAAS, to help you prepare publication quality figures: <https://journals.plos.org/plosone/s/figures#loc-tools-for-figure-preparation>.

NAAS will assess whether your figures meet our technical requirements by comparing each figure against our figure specifications.

*In compliance with data protection regulations, you may request that we remove your personal registration details at any time.* [*(Remove my information/details)*](https://track.editorialmanager.com/CL0/https:%2F%2Fwww.editorialmanager.com%2Fpone%2Flogin.asp%3Fa=r/1/010f019d945bb446-6969edb1-867a-4136-b18a-9166b80428f6-000000/ycttK9yUvzE-XlOZTofk7ubFjJ9fHJrErfL8cWP3SZ4=258)*. Please contact the publication office if you have any questions*
