# Supplementary material for: A phase III double-blind, placebo-controlled, randomized withdrawal trial of 5‑aminolevulinic acid hydrochloride with sodium ferrous citrate for efficacy and safety in patients diagnosed as Leigh syndrome
Source: PLoS One. 2026 Jul 17;21(7):e0332283. doi: 10.1371/journal.pone.0332283 (PMC13379092; doi:10.1371/journal.pone.0332283)
Supplement: Attachment — Submitted filename: Response to Reviewers PONE-D-25-46225-R3.docx [file pone.0332283.s024.docx]

RE: PONE-D-25-46225-R2

Authors’ Response to Reviewer #4:

Reviewer’s Comments on the second revised manuscript PONE-D-25-46225-R2

I have a couple of minor suggestions/comments:
1. The added text on sample size, heterogeneity and cautious interpretation remains primarily focused on efficacy. The manuscript still conveys a strong statement that the SAEs are unrelated to the study drug. In the absence of a clear explanation for the marked imbalance between SPP-004 and placebo group (64% vs 7%), this difference should be more explicitly and transparently acknowledged in the Results/Discussion section.
Authors’ Response to Reviewer ＃４>> The adverse events for the open-label period and the DB-period (SAS) were shown in Table 5, which indicated the marked imbalance between SPP-004 and placebo group (64% vs 7%) as pointed out by Reviewer #4. The patients allocated to SPP-004 by randomization had more advanced conditions with higher NPMDS scores compared to those of Placebo group as shown in Supplemental Table 3 and Supplemental Figure 1B. Therefore, their symptoms associated with the disease itself were observed more often in the patients of SP-004 compared to those of Placebo. Therefore, we concluded the SAE observed were unrelated to SP-004. However, this statement doesn’t sound fair as pointed out by Reviewer #4. We should tone down our statement and now describe as follows.

“The SAEs appeared to be unrelated to the study drug, and the outcome was recovery. The SAE symptoms observed in the safety assessment were considered more likely to be associated with the underlying disease rather than the study drug.”

Accordingly, we revised our descriptions regarding Safety in the Results (Lines 541-543, Lines 546-550) and Discussion (Lines 665-670).
2. The supplemental figure 1- B shows the NPMDS total score for placebo arm at 48 weeks to be close to 32 while supplemental table 3 reports it to be 38.9. Please address the discrepancy and update the figure 1-A accordingly, if needed.

Authors’ Response to Reviewer ＃４>> We are thankful to the reviewer for pointing out a critical discrepancy of the data shown in Supplemental Figure 1B and Supplemental Table 3. The data shown in Supplemental Table 3 are correct. We inadvertently picked up the incorrect Supplemental Figure 1B from our CSR. We noticed that the data plotted in the previous Supplemental Figure 1B were not adjusted by LOCF. The lower value at week 48 in the previous figure without LOCF is likely attributable to differential dropout in the placebo group, where patients with higher NPMDS scores tended to discontinue earlier, leaving patients with relatively lower scores at later time points.

Therefore, we corrected Supplemental Figure 1B with the data adjusted by LOCF, which were identical to those shown in Supplemental Table 3.
