# Supplementary material for: A phase III double-blind, placebo-controlled, randomized withdrawal trial of 5‑aminolevulinic acid hydrochloride with sodium ferrous citrate for efficacy and safety in patients diagnosed as Leigh syndrome
Source: PLoS One. 2026 Jul 17;21(7):e0332283. doi: 10.1371/journal.pone.0332283 (PMC13379092; doi:10.1371/journal.pone.0332283)
Supplement: Peer review history

# Peer review: Decision Letter 0

**DOI:** 10.1371/journal.pone.0332283.r001

**Authors:** Mehran Rahimlou

20 Nov 2025

PONE-D-25-46225

A phase III double-blind, placebo-controlled, randomized withdrawal trial of 5‑aminolevulinic acid hydrochloride with sodium ferrous citrate for efficacy and safety in patients diagnosed as Leigh syndrome

PLOS ONE

Dear Dr.

Nakajima,

Thank you for submitting your manuscript to PLOS ONE. After careful consideration, we feel that it has merit but does not fully meet PLOS ONE’s publication criteria as it currently stands. Therefore, we invite you to submit a revised version of the manuscript that addresses the points raised during the review process.

Please submit your revised manuscript by Jan 04 2026 11:59PM. If you will need more time than this to complete your revisions, please reply to this message or contact the journal office at plosone@plos.org. When you're ready to submit your revision, log on to https://www.editorialmanager.com/pone/ and select the 'Submissions Needing Revision' folder to locate your manuscript file.

Please include the following items when submitting your revised manuscript:

- A rebuttal letter that responds to each point raised by the academic editor and reviewer(s). You should upload this letter as a separate file labeled 'Response to Reviewers'.
- A marked-up copy of your manuscript that highlights changes made to the original version. You should upload this as a separate file labeled 'Revised Manuscript with Track Changes'.
- An unmarked version of your revised paper without tracked changes. You should upload this as a separate file labeled 'Manuscript'.

If you would like to make changes to your financial disclosure, please include your updated statement in your cover letter. Guidelines for resubmitting your figure files are available below the reviewer comments at the end of this letter.

If applicable, we recommend that you deposit your laboratory protocols in protocols.io to enhance the reproducibility of your results. Protocols.io assigns your protocol its own identifier (DOI) so that it can be cited independently in the future. For instructions see: https://journals.plos.org/plosone/s/submission-guidelines#loc-laboratory-protocols. Additionally, PLOS ONE offers an option for publishing peer-reviewed Lab Protocol articles, which describe protocols hosted on protocols.io. Read more information on sharing protocols at https://plos.org/protocols?utm_medium=editorial-email&utm_source=authorletters&utm_campaign=protocols.

We look forward to receiving your revised manuscript.

Kind regards,

Mehran Rahimlou, PhD

Academic Editor

PLOS ONE

Journal Requirements:

When submitting your revision, we need you to address these additional requirements.

1. Please ensure that your manuscript meets PLOS ONE's style requirements, including those for file naming. The PLOS ONE style templates can be found at

https://journals.plos.org/plosone/s/file?id=wjVg/PLOSOne_formatting_sample_main_body.pdf and

https://journals.plos.org/plosone/s/file?id=ba62/PLOSOne_formatting_sample_title_authors_affiliations.pdf

2. Thank you for stating the following in the Competing Interests/Financial Disclosure section:

We have read the journal's policy and the authors of this manuscript have the following competing interests: Hitoshi Nakagawa, Shunichi Kurose, Kaori Fukuda, Kiwamu Takahashi, Mitsugu Yamauchi, and Motowo Nakajima are employees of SBI Pharmaceuticals.

We note that one or more of the authors are employed by a commercial company: SBI Pharmaceuticals

a. Please provide an amended Funding Statement declaring this commercial affiliation, as well as a statement regarding the Role of Funders in your study. If the funding organization did not play a role in the study design, data collection and analysis, decision to publish, or preparation of the manuscript and only provided financial support in the form of authors' salaries and/or research materials, please review your statements relating to the author contributions, and ensure you have specifically and accurately indicated the role(s) that these authors had in your study. You can update author roles in the Author Contributions section of the online submission form.

Please also include the following statement within your amended Funding Statement.

“The funder provided support in the form of salaries for authors [insert relevant initials], but did not have any additional role in the study design, data collection and analysis, decision to publish, or preparation of the manuscript. The specific roles of these authors are articulated in the ‘author contributions’ section.”

If your commercial affiliation did play a role in your study, please state and explain this role within your updated Funding Statement.

b. Please also provide an updated Competing Interests Statement declaring this commercial affiliation along with any other relevant declarations relating to employment, consultancy, patents, products in development, or marketed products, etc.

Within your Competing Interests Statement, please confirm that this commercial affiliation does not alter your adherence to all PLOS ONE policies on sharing data and materials by including the following statement: "This does not alter our adherence to PLOS ONE policies on sharing data and materials.” (as detailed online in our guide for authors http://journals.plos.org/plosone/s/competing-interests) . If this adherence statement is not accurate and there are restrictions on sharing of data and/or materials, please state these. Please note that we cannot proceed with consideration of your article until this information has been declared.

Please include both an updated Funding Statement and Competing Interests Statement in your cover letter. We will change the online submission form on your behalf.

3. Please amend the manuscript submission data (via Edit Submission) to include author Kei Murayama

4. Please amend your list of authors on the manuscript to ensure that each author is linked to an affiliation. Authors’ affiliations should reflect the institution where the work was done (if authors moved subsequently, you can also list the new affiliation stating “current affiliation:….” as necessary).

5. We note that the original protocol file you uploaded contains a confidentiality notice indicating that the protocol may not be shared publicly or be published. Please note, however, that the PLOS Editorial Policy requires that the original protocol be published alongside your manuscript in the event of acceptance. Please note that should your paper be accepted, all content including the protocol will be published under the Creative Commons Attribution (CC BY) 4.0 license, which means that it will be freely available online, and any third party is permitted to access, download, copy, distribute, and use these materials in any way, even commercially, with proper attribution.

Therefore, we ask that you please seek permission from the study sponsor or body imposing the restriction on sharing this document to publish this protocol under CC BY 4.0 if your work is accepted. We kindly ask that you upload a formal statement signed by an institutional representative clarifying whether you will be able to comply with this policy. Additionally, please upload a clean copy of the protocol with the confidentiality notice (and any copyrighted institutional logos or signatures) removed.

6. If the reviewer comments include a recommendation to cite specific previously published works, please review and evaluate these publications to determine whether they are relevant and should be cited. There is no requirement to cite these works unless the editor has indicated otherwise.

[Note: HTML markup is below. Please do not edit.]

Reviewers' comments:

Reviewer's Responses to Questions

**Comments to the Author**

1. Is the manuscript technically sound, and do the data support the conclusions?

The manuscript must describe a technically sound piece of scientific research with data that supports the conclusions. Experiments must have been conducted rigorously, with appropriate controls, replication, and sample sizes. The conclusions must be drawn appropriately based on the data presented.

Reviewer #1: Yes

Reviewer #2: Yes

Reviewer #3: Partly

**********

2. Has the statistical analysis been performed appropriately and rigorously?

Reviewer #1: No

Reviewer #2: Yes

Reviewer #3: No

**********

3. Have the authors made all data underlying the findings in their manuscript fully available?

The PLOS Data policy requires authors to make all data underlying the findings described in their manuscript fully available without restriction, with rare exception (please refer to the Data Availability Statement in the manuscript PDF file). The data should be provided as part of the manuscript or its supporting information, or deposited to a public repository. For example, in addition to summary statistics, the data points behind means, medians and variance measures should be available. If there are restrictions on publicly sharing data—e.g. participant privacy or use of data from a third party—those must be specified.

Reviewer #1: Yes

Reviewer #2: Yes

Reviewer #3: Yes

**********

4. Is the manuscript presented in an intelligible fashion and written in standard English?

PLOS ONE does not copyedit accepted manuscripts, so the language in submitted articles must be clear, correct, and unambiguous. Any typographical or grammatical errors should be corrected at revision, so please note any specific errors here.

Reviewer #1: Yes

Reviewer #2: Yes

Reviewer #3: Yes

**********

5. Review Comments to the Author

Please use the space provided to explain your answers to the questions above. You may also include additional comments for the author, including concerns about dual publication, research ethics, or publication ethics. (Please upload your review as an attachment if it exceeds 20,000 characters)

Reviewer #1: Thank you for the opportunity to review this manuscript reporting the results of a Phase 3 trial in LS. Conducting a clinical trial in a rare pediatric disease such as LS is an extremely challenging and admirable undertaking. I commend the authors for their significant efforts in designing, executing, and reporting this important study.

Overall, the manuscript is well written and provides valuable data for the LS clinical and research community. The study appears to have been well executed, and the reporting is generally clear and comprehensive. However, I have several concerns regarding the analysis and interpretation of the results that I believe need to be addressed to strengthen the manuscript. My detailed comments are as follows:

Major Comments

Patient Discontinuations During the Open-Label (OL) Period (Lines 285–291)

According to the manuscript, 26 patients discontinued during the OL period. This represents a relatively high dropout rate. Based on the described study criteria, it appears these patients did not experience any clinical improvement during the OL phase.

Could the authors provide more detail on the reasons for these discontinuations?

If approximately half of the patients did not improve during the OL period, it raises questions about the interpretation of the positive results seen in the double-blind (DB) period. A deeper discussion of this point would be very helpful to readers in understanding the true clinical benefit.

Patient Withdrawal from the SPP and Inclusion in Analysis Sets

The manuscript notes that one patient withdrew from the SPP.

Please clarify the reason for this withdrawal and why this patient was excluded from the Full Analysis Set (FAS).

Given the small sample size of the study, it is critical to be cautious when excluding patients, especially from the FAS, as this could introduce bias and affect the robustness of the results.

I strongly recommend performing a sensitivity analysis that includes this patient to evaluate whether the study’s findings remain consistent.

Since this patient was included in the Safety Analysis Set (SAS), inclusion in the efficacy analysis would provide a more complete picture of the study results and improve transparency.

Clarity of Figure 3 Legend

In Figure 3, the placebo group includes a symbol labeled “Mb.” However, this symbol is not defined in the figure legend. Please clarify what “Mb” represents to ensure the figure is fully interpretable.

High Rate of Upper Respiratory Events and Infections (SAEs)

The manuscript reports a relatively high frequency of upper respiratory events and infections categorized as Serious Adverse Events (SAEs).

Could the authors provide potential explanations for these findings?

It would be helpful to know whether these events were considered related to the study drug, underlying disease, or external factors (e.g., seasonal illness).

A brief discussion on how these safety signals compare to similar studies in this patient population would provide valuable context for clinicians and researchers.

Context of Current Standard Clinical Interventions (Introduction, Line 110)

In the introduction, the authors state that there are currently no approved drugs for LS. While this is important to note, it would also be helpful to provide a brief overview of current standard non-pharmacological clinical interventions or management strategies used to alleviate symptoms. This additional context would help readers better understand the clinical landscape and unmet medical need that this trial addresses.

General Comments

While the study design and execution are commendable, certain aspects of the analysis and interpretation lack clarity. Addressing the points above will significantly improve the transparency and robustness of the manuscript.

Given the small sample size and challenges inherent in rare disease trials, it is especially important to ensure that all decisions regarding patient inclusion/exclusion and data interpretation are fully justified and clearly communicated.

I encourage the authors to include additional details in the Discussion to help readers better understand the implications of the study’s findings for both clinical practice and future research.

Summary Recommendation

This is a meaningful and well-conducted Phase 3 study in a rare pediatric population. The authors should be commended for their efforts to advance research and treatment options in LS. With revisions to address the concerns noted above, I believe this manuscript has the potential to make a strong contribution to the literature.

I would be pleased to review a revised version once these issues have been addressed.

Thank you again for the opportunity to review this important work.

Reviewer #2: This is an automated report for PONE-D-25-46225. This report was solicited by the PLOS One editorial team and provided by ScreenIT.

ScreenIT is an independent group of scientists developing automated tools that analyze academic papers. A set of automated tools screened your submitted manuscript and provided the report below. Each tool was created by your academic colleagues with the goal of helping authors. The tools look for factors that are important for transparency, rigor and reproducibility, and we hope that the report might help you to improve reporting in your manuscript. Within the report you will find links to more information about the items that the tools check. These links include helpful papers, websites, or videos that explain why the item is important. While our screening tools aim to improve and maintain quality standards they may, on occasion, miss nuances specific to your study type or flag something incorrectly. Each tool has limitations that are described on the ScreenIT website. The tools screen the main file for the paper; they are not able to screen supplements stored in separate files. Please note that the Academic Editor had access to these comments while making a decision on your manuscript. The Academic Editor may ask that issues flagged in this report be addressed. If you would like to provide feedback on the ScreenIT tool, please email the team at ScreenIt@bih-charite.de. If you have questions or concerns about the review process, please contact the PLOS One office at plosone@plos.org.

Reviewer #3: This manuscript presents data analysis from a Phase III, double-blind, placebo-controlled, randomized withdrawl trial to assess the efficiacy and safety of SPP-004 in patients with Leigh syndrome exhibiting CNS disorders. The topic is of importance, and the study was registered as a RCT within the Japanese Clinical Trial registry (with a valid jRCT number), and was approved by the respective IRB/Ethics Committee. While the study objectives sound interesting, is important, and on target, some shortcomings were observed in regards to abiding by the CONSORT guidelines for conducting and reporting results of high-quality randomized controlled trials.

1. Methods:

Methods reporting need some work. An orderly manner is suggested, following CONSORT guidelines, without repeating information, such as Trial Design, Participant Eligibility Criteria and settings, Interventions, Outcomes, sample size/power considerations, Interim analysis and stopping rules, Randomization (details on random number generation, allocation concealment, implementation), Blinding issues, etc, should be mentioned. The authors are advised to create "separate subsections" for each of the possible topics (whichever necessary), and that way produce a very clear writeup. They are advised to write it carefully, following nice examples in the manuscript below:

https://www.sciencedirect.com/science/article/pii/S0889540619300010

Specific comments:

(a) For instance, the randomization and allocation concealment should be made very clear (they are NOT the same thing); the trial staff recruiting patients should NOT have the randomization list. Randomization should be prepared by the trial statistician, and he/she would not participate in the recruiting.

(b) More details on the randomization is needed; I could find no such details. Was it a block randomization? What block size?

(c) Sample size: The sample size/power statement should mention the "name of the specific test used", and the corresponding effect size desired. Also, a separate paragraph would serve the readers better.

(d) Statistical Analysis writeup

(d1) The statistical analysis writeup proposes LOCFs to impute missing values (from early discontinuation). That the LOCF method has significant limitations is already noted in the literature. Is it possible for the authors to assess the sensitivity of this LOCF-based imputation, to something like multiple imputation technique, often implemented via the MICE algorithm; see here: https://www.jstatsoft.org/article/view/v045i03

(d2) The statistical methods presented are restricted to only finding confidence interval for the ratio, using Clopper-Pearson method. I believe, during the trial, various subject-level covariates were also collected. Was there any particular reason, why a subsequent regression approach was not considered, and statistical analysis only restricted to finding ratios and their confidence intervals?

(d3) Kaplan-Meier methods were used (Figure 2A), but those were never mentioned in the statistical analysis writeup.

2. Results & Conclusions:

(a) The authors should check that any statement of significance should be followed by a p-value in the entire Results section. Otherwise, the Results section look OK; it's pretty straightforward.

(b) Conclusions should state that the current findings are ONLY based on the samples derived from the Japanese population, and should allude to future studies with much larger sample sizes and collected at other geographical areas to confirm the effectiveness of the SPP-004.

**********

6. PLOS authors have the option to publish the peer review history of their article (what does this mean?). If published, this will include your full peer review and any attached files.

If you choose “no”, your identity will remain anonymous but your review may still be made public.

**Do you want your identity to be public for this peer review?**  For information about this choice, including consent withdrawal, please see our Privacy Policy.

Reviewer #1: **Yes:**  Ran Liao

Reviewer #2: No

Reviewer #3: No

**********

[NOTE: If reviewer comments were submitted as an attachment file, they will be attached to this email and accessible via the submission site. Please log into your account, locate the manuscript record, and check for the action link "View Attachments". If this link does not appear, there are no attachment files.]

To ensure your figures meet our technical requirements, please review our figure guidelines: https://journals.plos.org/plosone/s/figures

You may also use PLOS’s free figure tool, NAAS, to help you prepare publication quality figures: https://journals.plos.org/plosone/s/figures#loc-tools-for-figure-preparation.

NAAS will assess whether your figures meet our technical requirements by comparing each figure against our figure specifications.

Attachment Submitted filename: PLOS_ONE_LS_Ran_Comments.docx

Attachment Submitted filename: report_10.1101+2025.08.31.25334800.pdf

# Author comment: Author response to Decision Letter 1

**DOI:** 10.1371/journal.pone.0332283.r002

10 Dec 2025

Reply to Reviewer 1

Subject: Review of Manuscript – PONE-D-25-46225 - [EMID:9175fe2d96d786e0]

Dear Editor and Authors,

Thank you for the opportunity to review this manuscript reporting the results of a Phase 3 trial in LS. Conducting a clinical trial in a rare pediatric disease such as LS is an extremely challenging and admirable undertaking. I commend the authors for their significant efforts in designing, executing, and reporting this important study.

Overall, the manuscript is well written and provides valuable data for the LS clinical and research community. The study appears to have been well executed, and the reporting is generally clear and comprehensive. However, I have several concerns regarding the analysis and interpretation of the results that I believe need to be addressed to strengthen the manuscript. My detailed comments are as follows:

________________________________________

Major Comments

1. Patient Discontinuations During the Open-Label (OL) Period (Lines 285–291)

According to the manuscript, 26 patients discontinued during the OL period. This represents a relatively high dropout rate. Based on the described study criteria, it appears these patients did not experience any clinical improvement during the OL phase.

o Could the authors provide more detail on the reasons for these discontinuations?

The reason for withdrawal during the OL period was a lack of improvement in the NPMDS scores for cranial nervous symptoms and myopathy symptoms. To clarify the reasons for withdrawal, which were not previously stated, we have added this information on lines 290-292.

We distinguish between failure to transition from the OL period to the DB period as “withdrawal” and discontinuation during the DB period as “discontinuation”. In Table 1, Figure 1 and Figure 3, the terms were used incorrectly, so we have corrected them.

o If approximately half of the patients did not improve during the OL period, it raises questions about the interpretation of the positive results seen in the double-blind (DB) period. A deeper discussion of this point would be very helpful to readers in understanding the true clinical benefit.

As the reviewer pointed out, approximately half of the patients did not show improvement with SPP-004 administration during the OL period. However, as explained in the introduction, LS are genetically and biologically heterologous. Symptoms vary widely, and severity differs from patient to patient. Considering these factors, it is not surprising that approximately half of the cases did not show improvement during OL period. This idea is also supported by the fact that in vitro study using fibroblasts from MD patients by Shimura et al. (2019), improvement in ATP production or ORR was dependent on the gene mutations.

On the other hand, the efficacy in the DB phase represents a maintenance of the improvement achieved by SPP-004, that is, the effect maintained in patients for whom SPP-004 was effective during OL period. Therefore, we believe that the dropout of half of the patients during the OL period does not call into question the efficacy of SPP-004 in the DB period.

We have added a discussion on the dependence of the efficacy of SPP-004 on genetic mutations and symptoms in lines 562-571.

2. Patient Withdrawal from the SPP and Inclusion in Analysis Sets

The manuscript notes that one patient withdrew from the SPP.

o Please clarify the reason for this withdrawal and why this patient was excluded from the Full Analysis Set (FAS).

In this case, the SAE occurred immediately after the DB stage, so efficacy evaluation during the DB stage could never be performed. This has been added to the main text.

o Given the small sample size of the study, it is critical to be cautious when excluding patients, especially from the FAS, as this could introduce bias and affect the robustness of the results.

The comment of the reviewer was reasonable. However, we believe that the exclusion of the case was not arbitrary and does not pose any bias that would affect the robustness of the results.

o I strongly recommend performing a sensitivity analysis that includes this patient to evaluate whether the study’s findings remain consistent.

As mentioned above, there is no efficacy data for the DB period in this case, so it is not possible to include this in the efficacy analysis.

o Since this patient was included in the Safety Analysis Set (SAS), inclusion in the efficacy analysis would provide a more complete picture of the study results and improve transparency.

As mentioned above, there is no efficacy data for this case in the DB period, so it is considered appropriate to exclude it from the efficacy analysis. The validity of the study has been added to the revised manuscript, and the transparency of the research results is clear.

3. Clarity of Figure 3 Legend

In Figure 3, the placebo group includes a symbol labeled “Mb.” However, this symbol is not defined in the figure legend. Please clarify what “Mb” represents to ensure the figure is fully interpretable.

Thank you for pointing this out. The symbol and legend were inconsistent, so we have unified them both to Mb.

4. High Rate of Upper Respiratory Events and Infections (SAEs)

The manuscript reports a relatively high frequency of upper respiratory events and infections categorized as Serious Adverse Events (SAEs).

o Could the authors provide potential explanations for these findings?

We added the explanations of these SAEs in the discussions (Lines 574-

582).

o It would be helpful to know whether these events were considered related to the study drug, underlying disease, or external factors (e.g., seasonal illness).

We added the descriptions in the discussions. They are unrelated with the study drug and most events were due to accidental infections.

o A brief discussion on how these safety signals compare to similar studies in this patient population would provide valuable context for clinicians and researchers.

We added a brief discussion by citing a report by Sofou et al. (2014) that describes disease courses and predictors of survival in patients with LS.

5. Context of Current Standard Clinical Interventions (Introduction, Line 110)

In the introduction, the authors state that there are currently no approved drugs for LS. While this is important to note, it would also be helpful to provide a brief overview of current standard non-pharmacological clinical interventions or management strategies used to alleviate symptoms. This additional context would help readers better understand the clinical landscape and unmet medical need that this trial addresses.

In response to this comment, we have provided more details about clinical interventions and approved medications for LS (Lines 89-95 in the revised manuscript).

Reply to Reviewer 2: Screen IT Report

Rigor

Power analysis: Not detected

We explained the sample size limitation due to our difficulties in recruitment of very rare disease LS patients in Japan only. Further investigations with enough numbers of LS patients in different countries and areas are necessary for any power analysis.

Resources

Software and Algorithms: BioVendor suggested: BioVendor Laboratory Medicine (RRID:SCR_005143)

We could not confirm BioVenodr’s suggestion at their home page Immunoassays | BioVendor Research and Diagnostics Products. Therefore, we kept the product name in the methods instead of adding the suggested catalogue number (RRID:SCR_005143).

Transparency

Open Code and Open Data: If permitted, sharing data on a public open repository.

As mentioned in the manuscript, the clinical data and records are owned by each of the participants of the study and those are available on an appropriate official request from academic institutions for further investigation.

Reply to Reviewer 3

1. Methods:

Methods reporting need some work. An orderly manner is suggested, following CONSORT guidelines, without repeating information, such as Trial Design, Participant Eligibility Criteria and settings, Interventions, Outcomes, sample size/power considerations, Interim analysis and stopping rules, Randomization (details on random number generation, allocation concealment, implementation), Blinding issues, etc, should be mentioned. The authors are advised to create "separate subsections" for each of the possible topics (whichever necessary), and that way produce a very clear writeup. They are advised to write it carefully, following nice examples in the manuscript below:

https://www.sciencedirect.com/science/article/pii/S0889540619300010

We have revised the description in accordance with the Consort guidelines.

Specific comments:

(a) For instance, the randomization and allocation concealment should be made very clear (they are NOT the same thing); the trial staff recruiting patients should NOT have the randomization list. Randomization should be prepared by the trial statistician, and he/she would not participate in the recruiting.

We clearly described randomization and allocation concealment (lines 177-181). The randomization key code is managed by the Allocation manager, who is not involved in patient recruitment.

(b) More details on the randomization is needed; I could find no such details. Was it a block randomization? What block size?

We used the permuted block randomization method. Block size was not described because it was not disclosed.

(c) Sample size: The sample size/power statement should mention the "name of the specific test used", and the corresponding effect size desired. Also, a separate paragraph would serve the readers better.

We describe the sample size and effective size on lines 182-189.

(d) Statistical Analysis writeup

(d1) The statistical analysis writeup proposes LOCFs to impute missing values (from early discontinuation). That the LOCF method has significant limitations is already noted in the literature. Is it possible for the authors to assess the sensitivity of this LOCF-based imputation, to something like multiple imputation technique, often implemented via the MICE algorithm; see here: https://www.jstatsoft.org/article/view/v045i03

We understand the limitations of the LOCF method. However, if NPMDS scores are not adjusted, discrepancies may occur, such as an improvement in the mean score due to the dropout of patients whose symptoms worsened.

Adjustment using the Mice algorithm was not performed, as we were unaware of this method at the time of planning this study.

(d2) The statistical methods presented are restricted to only finding confidence interval for the ratio, using Clopper-Pearson method. I believe, during the trial, various subject-level covariates were also collected. Was there any particular reason, why a subsequent regression approach was not considered, and statistical analysis only restricted to finding ratios and their confidence intervals?

As you pointed out, various subject-level covariates were also collected during the trial. However, the study had a small sample size of 15 cases in each group at the time of planning, which was deemed insufficient for regression analysis. Therefore, we did not perform regression analysis.

(d3) Kaplan-Meier methods were used (Figure 2A), but those were never mentioned in the statistical analysis writeup.

We described Kaplan-Meier analysis in lines 225-228. Statistical comparison was performed by log-rank test as described in lines 229-230.

2. Results & Conclusions:

(a) The authors should check that any statement of significance should be followed by a p-value in the entire Results section. Otherwise, the Results section look OK; it's pretty straightforward.

We confirmed that p-values were reported for items tested for significance.

(b) Conclusions should state that the current findings are ONLY based on the samples derived from the Japanese population and should allude to future studies with much larger sample sizes and collected at other geographical areas to confirm the effectiveness of the SPP-004.

In response to the comments, the conclusion section now includes a discussion of future studies

Attachment Submitted filename: Response to Reviewers_PONE-D-25-46225.doc

# Peer review: Decision Letter 1

**DOI:** 10.1371/journal.pone.0332283.r003

**Authors:** Bhanwar Lal Puniya

15 Apr 2026

PONE-D-25-46225R1

A phase III double-blind, placebo-controlled, randomized withdrawal trial of 5‑aminolevulinic acid hydrochloride with sodium ferrous citrate for efficacy and safety in patients diagnosed as Leigh syndrome

PLOS One

Dear Dr. Nakajima,

Thank you for submitting your manuscript to PLOS ONE. After careful consideration, we feel that it has merit but does not fully meet PLOS ONE’s publication criteria as it currently stands. Therefore, we invite you to submit a revised version of the manuscript that addresses the points raised during the review process.

=======================================

The manuscript has clearly benefited from the revisions made in response to the previous reviewers. The manuscript has now been evaluated by an additional reviewer, whose comments are enclosed and should be addressed in detail, in addition to those from the previous reviewers.  In addition to those points, I have also listed my concerns that need further clarification before the manuscript can be considered for publication.

=======================================

Please submit your revised manuscript by May 30 2026 11:59PM. If you will need more time than this to complete your revisions, please reply to this message or contact the journal office at plosone@plos.org. When you're ready to submit your revision, log on to https://www.editorialmanager.com/pone/ and select the 'Submissions Needing Revision' folder to locate your manuscript file.

Please include the following items when submitting your revised manuscript:

- A letter that responds to each point raised by the academic editor and reviewer(s). You should upload this letter as a separate file labeled 'Response to Reviewers'.
- A marked-up copy of your manuscript that highlights changes made to the original version. You should upload this as a separate file labeled 'Revised Manuscript with Track Changes'.
- An unmarked version of your revised paper without tracked changes. You should upload this as a separate file labeled 'Manuscript'.

If you would like to make changes to your financial disclosure, please include your updated statement in your cover letter. Guidelines for resubmitting your figure files are available below the reviewer comments at the end of this letter.

If applicable, we recommend that you deposit your laboratory protocols in protocols.io to enhance the reproducibility of your results. Protocols.io assigns your protocol its own identifier (DOI) so that it can be cited independently in the future. For instructions see: https://journals.plos.org/plosone/s/submission-guidelines#loc-laboratory-protocols. Additionally, PLOS ONE offers an option for publishing peer-reviewed Lab Protocol articles, which describe protocols hosted on protocols.io. Read more information on sharing protocols at https://plos.org/protocols?utm_medium=editorial-email&utm_source=authorletters&utm_campaign=protocols.

As the corresponding author, your ORCID iD is verified in the submission system and will appear in the published article. PLOS supports the use of ORCID, and we encourage all coauthors to register for an ORCID iD and use it as well. Please encourage your coauthors to verify their ORCID iD within the submission system before final acceptance, as unverified ORCID iDs will not appear in the published article. *Only*  the individual author can complete the verification step; PLOS staff *cannot*  verify ORCID iDs on behalf of authors.

We look forward to receiving your revised manuscript.

Kind regards,

Bhanwar Lal Puniya, Ph.D.

Academic Editor

PLOS One

Journal Requirements:

1. If the reviewer comments include a recommendation to cite specific previously published works, please review and evaluate these publications to determine whether they are relevant and should be cited. There is no requirement to cite these works unless the editor has indicated otherwise.

Additional Editor Comments:

(1) In some sections, it is indicated that no specific funding was received. At the same time, in the cover letter it is stated that SBI Pharmaceuticals supported the study and provided salaries to some authors. Please revise the funding and competing interest sections, and clearly describe the role of the sponsor, if any, in study design, data collection, analysis, and manuscript preparation.

(2) Please check the data availability statements. At one point it is stated that all data are fully available, while elsewhere it is suggested that data are only available upon request. Please provide a clear data availability statement that is accurate and complies with journal policy.

(3) There needs to be a clearer presentation is the interpretation of the primary endpoint. As reported, the primary endpoint does not reach statistical significance, while the abstract and conclusions place more emphasis on positive findings from secondary analyses. The authors are encouraged to present a more balanced interpretation. It would help readers if the manuscript clearly states that the primary endpoint was not statistically significant and frames the other findings as supportive/exploratory, rather than definitive.

(4) Because only patients who improved during the open-label phase entered the randomized phase, the double-blind portion of the study evaluates maintenance of response in a selected group of responders.

This is a reasonable approach. However, it limits the generalizability of the findings. Please state this more explicitly (in the abstract and discussion), so that the scope of the conclusions is clear.

(5) The differences between groups in sex distribution and age at onset are noticeable because of small sample size. While this may be unavoidable, it would be helpful to acknowledge this more clearly as a limitation and add a comment on its possible impact on interpretation.

(6) The use of the LOCF method and the exclusion of one randomized patient from the efficacy analysis would benefit from additional clarification. In light of the additional reviewer’s comments, it would also be helpful to clarify how missing data and follow-up were handled, particularly regarding the optional Week-48 follow-up and the transition to the extension phase.

A brief clarification of (i) the rationale for using LOCF, (ii) its limitations, and (iii) how the analysis population was defined would improve transparency.

(7) A careful pass for language and clarity would help improve readability.

[Note: HTML markup is below. Please do not edit.]

Reviewer's Responses to Questions

**Comments to the Author**

1. If the authors have adequately addressed your comments raised in a previous round of review and you feel that this manuscript is now acceptable for publication, you may indicate that here to bypass the “Comments to the Author” section, enter your conflict of interest statement in the “Confidential to Editor” section, and submit your "Accept" recommendation.

Reviewer #1: All comments have been addressed

Reviewer #3: All comments have been addressed

Reviewer #4: (No Response)

**********

2. Is the manuscript technically sound, and do the data support the conclusions?

The manuscript must describe a technically sound piece of scientific research with data that supports the conclusions. Experiments must have been conducted rigorously, with appropriate controls, replication, and sample sizes. The conclusions must be drawn appropriately based on the data presented.

Reviewer #1: Yes

Reviewer #3: (No Response)

Reviewer #4: (No Response)

**********

3. Has the statistical analysis been performed appropriately and rigorously?

Reviewer #1: Yes

Reviewer #3: (No Response)

Reviewer #4: (No Response)

**********

4. Have the authors made all data underlying the findings in their manuscript fully available?

The PLOS Data policy requires authors to make all data underlying the findings described in their manuscript fully available without restriction, with rare exception (please refer to the Data Availability Statement in the manuscript PDF file). The data should be provided as part of the manuscript or its supporting information, or deposited to a public repository. For example, in addition to summary statistics, the data points behind means, medians and variance measures should be available. If there are restrictions on publicly sharing data—e.g. participant privacy or use of data from a third party—those must be specified.

Reviewer #1: No

Reviewer #3: (No Response)

Reviewer #4: (No Response)

**********

5. Is the manuscript presented in an intelligible fashion and written in standard English?

PLOS ONE does not copyedit accepted manuscripts, so the language in submitted articles must be clear, correct, and unambiguous. Any typographical or grammatical errors should be corrected at revision, so please note any specific errors here.

Reviewer #1: Yes

Reviewer #3: Yes

Reviewer #4: (No Response)

**********

6. Review Comments to the Author

Please use the space provided to explain your answers to the questions above. You may also include additional comments for the author, including concerns about dual publication, research ethics, or publication ethics. (Please upload your review as an attachment if it exceeds 20,000 characters)

Reviewer #1: Thank you to the author team for amending the manuscript and addressing my comments - I appreciate the effort. The manuscript has indeed improved considerably. I don't have further comments on the data itself, but I do have a concern about whether the author team has disclosed all data underlying the findings and made it fully available. I did not find any listing of patients with SAEs or patient narratives; the manuscript currently contains only summary-level tables and figures for the description of the data. I am fine with this approach if it aligns with PLOS data policy, but I wanted to point this out for clarification.

Reviewer #3: (No Response)

Reviewer #4: Summary of the Manuscript

The authors report a multicenter, double-blind, placebo-controlled, randomized withdrawal trial of an investigational agent targeting mitochondrial dysfunction in 54 patients aged 7.9 ± 6.52 with Leigh syndrome. The study included a 24-week open-label period followed by a 48-week double-blind period. The study was conducted between July 2018 and May 2020. The primary endpoint was the change from baseline in the Newcastle Pediatric Mitochondrial Disease Scale (NPMDS) score, section I and III, at Week 48. The primary efficacy endpoint was the proportion of patients discontinuing due to inadequate efficacy by Week 48. Secondary endpoints included the proportions of patients who discontinued due to inadequate efficacy at Weeks 24 and 36.

Major Strengths

This rare-disease trial in a challenging population is addressing a critical unmet need in mitochondrial medicine. I congratulate the authors for successfully combining an open-label lead-in with a randomized, double-blind, placebo-controlled phase, logistically difficult in progressive neurodegenerative disorders, while also monitoring both clinical and biomarker endpoints. Use of the NPMDS shows commitment to a disease-specific efficacy measure, and high retention during the open-label period shows strong operational execution. The manuscript is well-structured and thoughtfully incorporates multiple angles.

Questions, Concerns, and Suggestions

1. The protocol amendment made Week-48 follow-up optional for patients entering the long-term extension. Patients perceiving benefits are more likely to skip formal follow-up and enter extension early, leading to LOCF carrying forward last positive observations. This might inflate apparent efficacy. Please report the number of people who exercised the optional early-entry pathway and skipped mandatory follow-up.

2. The Exploratory trial that has been referred to (Abe et al., 2025) mentions “NPMDS section II and section III items 1 and 2 were not suitable for the evaluation of the results of these studies, as their substantial observation periods (3, 4, and 6 months) were longer than that of the study period (12 weeks for both the double- blind and open-label periods)”. However, the current study has a period of 24 weeks followed by 48 weeks (6 months and 12 months respectively).

Citing references/reasoning, if any, that support the modified subscale would be helpful. I strongly recommend reporting the full NPMDS total score as a key secondary endpoint, if available.

3. In untreated Leigh syndrome, natural history data show ~+2 points worsening on the full NPMDS over ~48 weeks (Lim et al. 2021). In your findings, on the modified subscale, placebo patients worsened on average from −1.9 ± 1.00 to −1.1 ± 1.83 (mean change +0.8), yet the large SD (±1.83) indicates that some individuals still showed improvement even on placebo. The SPP-004 group demonstrated a trend toward improvement (−1.6 ± 1.82 to −2.3 ± 2.18; mean −0.7).

Given the tiny between-group difference (~1.5 points) and high variability, pls comment on the robustness of the findings with respect to expected short-term variability of a slowly progressive condition. Pls consider adding full NPMDS data to add value.

Are there any clear differences between the placebo and SPP-004 groups baselines at the start of 48 weeks?

4. An imbalance in SAEs was observed during the double-blind period (64.3% [9/14] in the SPP-004 group vs 7.1% [1/14] in placebo). The authors attribute the high incidence of upper respiratory tract infections to incidental infections or underlying Leigh syndrome, and state they are not causally related to the study drug. However, if these events are due to incidental infections or disease progression, the incidence should have been comparable between arms, especially since the placebo group showed disease worsening. Clarity on how causality was formally assessed and whether any SAEs were considered “possibly” or “probably” related to the drug would be helpful.

**********

7. PLOS authors have the option to publish the peer review history of their article (what does this mean?). If published, this will include your full peer review and any attached files.

If you choose “no”, your identity will remain anonymous but your review may still be made public.

**Do you want your identity to be public for this peer review?**  For information about this choice, including consent withdrawal, please see our Privacy Policy.

Reviewer #1: **Yes:**  Ran Liao

Reviewer #3: No

Reviewer #4: No

**********

[NOTE: If reviewer comments were submitted as an attachment file, they will be attached to this email and accessible via the submission site. Please log into your account, locate the manuscript record, and check for the action link "View Attachments". If this link does not appear, there are no attachment files.]

To ensure your figures meet our technical requirements, please review our figure guidelines: https://journals.plos.org/plosone/s/figures

You may also use PLOS’s free figure tool, NAAS, to help you prepare publication quality figures: https://journals.plos.org/plosone/s/figures#loc-tools-for-figure-preparation.

NAAS will assess whether your figures meet our technical requirements by comparing each figure against our figure specifications.

# Author comment: Author response to Decision Letter 2

**DOI:** 10.1371/journal.pone.0332283.r004

13 May 2026

Additional Editor Comments:

(1) In some sections, it is indicated that no specific funding was received. At the same time, in the cover letter it is stated that SBI Pharmaceuticals supported the study and provided salaries to some authors. Please revise the funding and competing interest sections, and clearly describe the role of the sponsor, if any, in study design, data collection, analysis, and manuscript preparation.

>>Answer to Editor: According to the editor’s advice the role of the sponsor, SBI Pharmaceuticals, was clearly described in the declaration of Competing Interest. The roles of the employees of SBI Pharmaceuticals were shown in the co-authors’ contributions.

(2) Please check the data availability statements. At one point it is stated that all data are fully available, while elsewhere it is suggested that data are only available upon request. Please provide a clear data availability statement that is accurate and complies with journal policy.　>> Answer to Editor: Thank you for your advice. We made our descriptions of data availability consistent throughout the manuscript. All the data is fully available (lines 723-725).

(3) There needs to be a clearer presentation is the interpretation of the primary endpoint. As reported, the primary endpoint does not reach statistical significance, while the abstract and conclusions place more emphasis on positive findings from secondary analyses. The authors are encouraged to present a more balanced interpretation. It would help readers if the manuscript clearly states that the primary endpoint was not statistically significant and frames the other findings as supportive/exploratory, rather than definitive.

>> Answer to Editor: We carefully checked our conclusions in Abstract and Discussion and clearly stated that the primary endpoint was not statistically significant and the other findings were framed as supportive/exploratory, rather than definitive.

(4) Because only patients who improved during the open-label phase entered the randomized phase, the double-blind portion of the study evaluates maintenance of response in a selected group of responders.

This is a reasonable approach. However, it limits the generalizability of the findings. Please state this more explicitly (in the abstract and discussion), so that the scope of the conclusions is clear.

>> Answer to Editor:

We thank the reviewer for this constructive comment. We agree that, because only patients who showed clinical improvement during the open label phase were randomized, the double blind phase evaluates maintenance of response in a responder enriched population, which limits generalizability.

To address this, we have revised the Abstract (lines 46-53, lines 61-62) to explicitly state that the study objective and conclusions concern maintenance of therapeutic benefit in patients who achieved an initial response. We have also revised the Discussion to clearly acknowledge this design feature as a limitation and to note that the findings should be interpreted within this context (line 589-600).

(5) The differences between groups in sex distribution and age at onset are noticeable because of small sample size. While this may be unavoidable, it would be helpful to acknowledge this more clearly as a limitation and add a comment on its possible impact on interpretation.

>> Answer to Editor: Although the differences between groups in sex distribution and age at onset were noticeable, there is a limitation of its possible impact on interpretation due to small sample size. This limitation has now been more clearly stated in the Discussion (lines 659-668), together with an explanation that recruitment of a larger and more balanced cohort was challenging because Leigh syndrome is a rare disease.

(6) The use of the LOCF method and the exclusion of one randomized patient from the efficacy analysis would benefit from additional clarification. In light of the additional reviewer’s comments, it would also be helpful to clarify how missing data and follow-up were handled, particularly regarding the optional Week-48 follow-up and the transition to the extension phase.

A brief clarification of (i) the rationale for using LOCF, (ii) its limitations, and (iii) how the analysis population was defined would improve transparency.

>> Answer to Editor:

We thank the reviewer for this helpful comment. We have revised the Statistical analysis section to clarify the rationale for using the last observation carried forward (LOCF) method, the handling of missing data and follow up, and the definition of the analysis population. Specifically, we now explain that LOCF was used to handle missing efficacy data due to early discontinuation and to support consistent longitudinal comparisons (lines 280-283). We also clarify that when the Week 48 follow up was not completed because of early discontinuation or transition to the extension study, efficacy was evaluated using the last available assessment obtained at discontinuation or prior to transition (lines 284–286).

Furthermore, we have already described that the double blind efficacy analysis population (FAS) included all randomized patients with at least one post randomization efficacy assessment, and that one randomized patient was excluded due to the absence of any post randomization efficacy data following an early serious adverse event (lines 299-301). A brief statement acknowledging the limitations of the LOCF approach has also been added to the Discussion (lines 666–668).

(7) A careful pass for language and clarity would help improve readability.

>> Answer to Editor: The additional editorial work was performed by a native English scientist to improve readability.

Review Comments to the Author

Please use the space provided to explain your answers to the questions above. You may also include additional comments for the author, including concerns about dual publication, research ethics, or publication ethics. (Please upload your review as an attachment if it exceeds 20,000 characters)

Reviewer #1: Thank you to the author team for amending the manuscript and addressing my comments - I appreciate the effort. The manuscript has indeed improved considerably. I don't have further comments on the data itself, but I do have a concern about whether the author team has disclosed all data underlying the findings and made it fully available. I did not find any listing of patients with SAEs or patient narratives; the manuscript currently contains only summary-level tables and figures for the description of the data. I am fine with this approach if it aligns with PLOS data policy, but I wanted to point this out for clarification.

Answer to Reviewer #2:

In the table of SAE (S10 table) in the open label and double-blind periods, not only the number of patients but also their IDs were included in each SAE case. A few patients showed more than 2 SAEs, however, these were often related to infections. We included these data and observations in the results.

Reviewer #3: (No Response)

Reviewer #4: Summary of the Manuscript

The authors report a multicenter, double-blind, placebo-controlled, randomized withdrawal trial of an investigational agent targeting mitochondrial dysfunction in 54 patients aged 7.9 ± 6.52 with Leigh syndrome. The study included a 24-week open-label period followed by a 48-week double-blind period. The study was conducted between July 2018 and May 2020. The primary endpoint was the change from baseline in the Newcastle Pediatric Mitochondrial Disease Scale (NPMDS) score, section I and III, at Week 48. The primary efficacy endpoint was the proportion of patients discontinuing due to inadequate efficacy by Week 48. Secondary endpoints included the proportions of patients who discontinued due to inadequate efficacy at Weeks 24 and 36.

Major Strengths

This rare-disease trial in a challenging population is addressing a critical unmet need in mitochondrial medicine. I congratulate the authors for successfully combining an open-label lead-in with a randomized, double-blind, placebo-controlled phase, logistically difficult in progressive neurodegenerative disorders, while also monitoring both clinical and biomarker endpoints. Use of the NPMDS shows commitment to a disease-specific efficacy measure, and high retention during the open-label period shows strong operational execution. The manuscript is well-structured and thoughtfully incorporates multiple angles.

Questions, Concerns, and Suggestions

1. The protocol amendment made Week-48 follow-up optional for patients entering the long-term extension. Patients perceiving benefits are more likely to skip formal follow-up and enter extension early, leading to LOCF carrying forward last positive observations. This might inflate apparent efficacy. Please report the number of people who exercised the optional early-entry pathway and skipped mandatory follow-up.

>> Answer to Reviewer #4:

We thank the reviewer for raising this important point regarding the potential impact of the protocol amendment.

All patients who entered the long term extension without completing the　48-week DB-period (n=9) discontinued the double blind study due to insufficient efficacy; no early entry was driven by perceived clinical benefit. Thus, the concern that favorable outcomes were preferentially carried forward by LOCF is not supported by the data. This has been explained in the result section (lines 304-306).

In addition, the amendment making the follow up (at 48-week or at time of discontinuation) optional was implemented from a patient protection perspective, with the intention of minimizing the treatment free period after discontinuation for insufficient efficacy, rather than to selectively advance patients with favorable responses.

2. The Exploratory trial that has been referred to (Abe et al., 2025) mentions “NPMDS section II and section III items 1 and 2 were not suitable for the evaluation of the results of these studies, as their substantial observation periods (3, 4, and 6 months) were longer than that of the study period (12 weeks for both the double- blind and open-label periods)”. However, the current study has a period of 24 weeks followed by 48 weeks (6 months and 12 months respectively).

Citing references/reasoning, if any, that support the modified subscale would be helpful. I strongly recommend reporting the full NPMDS total score as a key secondary endpoint, if available.

>> Answer to Reviewer #4:

We thank the reviewer for the important suggestion regarding inclusion of full NPMDS data.

In response, we have added the full NPMDS longitudinal data as Supplemental Figure 1. As expected, the full NPMDS demonstrated greater inter individual variability than the modified NPMDS for cranial nervous symptoms and myopathy symptoms (Lines 380-391), reflecting the heterogeneous and slowly progressive nature of Leigh syndrome.

Despite this increased variability, a trend toward maintenance of improvement was more frequently observed in the SPP 004 group, whereas placebo treated patients showed greater fluctuation and progression over the 48 week double blind period. These findings are consistent with the primary analysis and support the interpretation that SPP 004 may contribute to stabilization rather than short term symptomatic change.

We have also clarified in the Discussion that, due to the limited sensitivity of several full NPMDS domains over short term follow up, these results should be interpreted cautiously and primarily as supportive evidence (lines 589-600).

3. In untreated Leigh syndrome, natural history data show ~+2 points worsening on the full NPMDS over ~48 weeks (Lim et al. 2021). In your findings, on the modified subscale, placebo patients worsened on average from −1.9 ± 1.00 to −1.1 ± 1.83 (mean change +0.8), yet the large SD (±1.83) indicates that some individuals still showed improvement even on placebo. The SPP-004 group demonstrated a trend toward improvement (−1.6 ± 1.82 to −2.3 ± 2.18; mean −0.7).

Given the tiny between-group difference (~1.5 points) and high variability, pls comment on the robustness of the findings with respect to expected short-term variability of a slowly progressive condition. Pls consider adding full NPMDS data to add value.

Are there any clear differences between the placebo and SPP-004 groups baselines at the start of 48 weeks?

>> Answer to Reviewer #4:

We thank the reviewer for highlighting the issue of baseline comparability.

As shown in Supplemental Table 3, patients in the SPP-004 group had higher full NPMDS scores at the start of the double-blind period (mean ± SD: 50.3 ± 20.1) compared with the placebo group (40.9 ± 19.4), indicating a greater baseline disease severity in the SPP-004 group.

This imbalance suggests that patients randomized to SPP-004 were, on average, more severely affected at baseline. It was a factor that would be expected to bias outcomes toward less improvement rather than favoring an apparent treatment effect. Despite this disadvantage, the SPP-004 group demonstrated overall maintenance or improvement in both the modified NPMDS subscale and the full NPMDS score during the double-blind period, whereas the placebo group showed a tendency toward stabilization or mild worsening.

Given the progressive nature of Leigh syndrome and the approximately +2-point deterioration over 48 weeks reported in untreated patients in natural history studies (Lim et al., 2021), the observed trajectories suggest that SPP-004 may have attenuated disease progression even in a more severely affected population.

We acknowledge that the small sample size, baseline imbalance, and substantial inter-individual variability limit definitive conclusions. Accordingly, these findings should be interpreted as supportive rather than confirmatory evidence of treatment benefit. We have revised the Results (lines 380-391) and Discussion sections (lines 589-600) to explicitly address this baseline difference and its implications for robustness and interpretation.

4. An imbalance in SAEs was observed during the double-blind period (64.3% [9/14] in the SPP-004 group vs 7.1% [1/14] in placebo). The authors attribute the high incidence of upper respiratory tract infections to incidental infections or underlying Leigh syndrome, and state they are not causally related to the study drug. However, if these events are due to incidental infections or disease progression, the incidence should have been comparable between arms, especially since the placebo group showed disease worsening. Clarity on how causality was formally assessed and whether any SAEs were considered “possibly” or “probably” related to the drug would be helpful.

>> Answer to Reviewer #4:

We thank the reviewer for this important comment regarding the numerical imbalance in serious adverse events (SAEs) observed during the double-blind period.

All SAEs were formally assessed for causality by the investigators in accordance with the study protocol and Good Clinical Practice criteria. As a result of this systematic assessment, none of the reported SAEs, including those occurring during the double-blind period, was considered possibly or probably related to SPP-004.

To improve clarity, we have revised the Results section to explicitly state that all SAEs were judged as not related to the study drug. In addition, Table 5 has been updated to include a specific category for serious adverse drug reactions (serious adverse reactions), clearly demonstrating that no serious adverse reactions were observed in either treatment group.

Furthermore, we have revised the Discussion section to acknowledge the numerical imbalance in SAE incidence between treatment arms and to clarify that this imbalance should be interpreted with caution, given the small sample size, baseline heterogeneity, and the well-recognized susceptibility of patients with Leigh syndrome to intercurrent infections independent of study treatment.

These clarifications have been added to better address the reviewer’s concern and to strengthen the interpretation of the safety findings.

Attachment Submitted filename: Responces to reviewers_PONE-D-25-46225-R2.docx

# Peer review: Decision Letter 2

**DOI:** 10.1371/journal.pone.0332283.r005

**Authors:** Bhanwar Lal Puniya

20 May 2026

PONE-D-25-46225R2

A phase III double-blind, placebo-controlled, randomized withdrawal trial of 5‑aminolevulinic acid hydrochloride with sodium ferrous citrate for efficacy and safety in patients diagnosed as Leigh syndrome

PLOS One

Dear Dr. Nakajima,

Thank you for submitting your manuscript to PLOS ONE. After careful consideration, we feel that it has merit but does not fully meet PLOS ONE’s publication criteria as it currently stands. Therefore, we invite you to submit a revised version of the manuscript that addresses the points raised during the review process.

Please address the minor comments by Reviewer #4.

Please submit your revised manuscript by Jul 04 2026 11:59PM. If you will need more time than this to complete your revisions, please reply to this message or contact the journal office at plosone@plos.org. When you're ready to submit your revision, log on to https://www.editorialmanager.com/pone/ and select the 'Submissions Needing Revision' folder to locate your manuscript file.

Please include the following items when submitting your revised manuscript:

- A letter that responds to each point raised by the academic editor and reviewer(s). You should upload this letter as a separate file labeled 'Response to Reviewers'.
- A marked-up copy of your manuscript that highlights changes made to the original version. You should upload this as a separate file labeled 'Revised Manuscript with Track Changes'.
- An unmarked version of your revised paper without tracked changes. You should upload this as a separate file labeled 'Manuscript'.

If you would like to make changes to your financial disclosure, please include your updated statement in your cover letter. Guidelines for resubmitting your figure files are available below the reviewer comments at the end of this letter.

If applicable, we recommend that you deposit your laboratory protocols in protocols.io to enhance the reproducibility of your results. Protocols.io assigns your protocol its own identifier (DOI) so that it can be cited independently in the future. For instructions see: https://journals.plos.org/plosone/s/submission-guidelines#loc-laboratory-protocols. Additionally, PLOS ONE offers an option for publishing peer-reviewed Lab Protocol articles, which describe protocols hosted on protocols.io. Read more information on sharing protocols at https://plos.org/protocols?utm_medium=editorial-email&utm_source=authorletters&utm_campaign=protocols.

As the corresponding author, your ORCID iD is verified in the submission system and will appear in the published article. PLOS supports the use of ORCID, and we encourage all coauthors to register for an ORCID iD and use it as well. Please encourage your coauthors to verify their ORCID iD within the submission system before final acceptance, as unverified ORCID iDs will not appear in the published article. *Only*  the individual author can complete the verification step; PLOS staff *cannot*  verify ORCID iDs on behalf of authors.

We look forward to receiving your revised manuscript.

Kind regards,

Bhanwar Lal Puniya, Ph.D.

Academic Editor

PLOS One

**Journal Requirements:**

1. If the reviewer comments include a recommendation to cite specific previously published works, please review and evaluate these publications to determine whether they are relevant and should be cited. There is no requirement to cite these works unless the editor has indicated otherwise.

2. Please review your reference list to ensure that it is complete and correct. If you have cited papers that have been retracted, please include the rationale for doing so in the manuscript text, or remove these references and replace them with relevant current references. Any changes to the reference list should be mentioned in the rebuttal letter that accompanies your revised manuscript. If you need to cite a retracted article, indicate the article’s retracted status in the References list and also include a citation and full reference for the retraction notice.

[Note: HTML markup is below. Please do not edit.]

Reviewers' comments:

Reviewer's Responses to Questions

**Comments to the Author**

1. If the authors have adequately addressed your comments raised in a previous round of review and you feel that this manuscript is now acceptable for publication, you may indicate that here to bypass the “Comments to the Author” section, enter your conflict of interest statement in the “Confidential to Editor” section, and submit your "Accept" recommendation.

Reviewer #4: (No Response)

**********

2. Is the manuscript technically sound, and do the data support the conclusions?

The manuscript must describe a technically sound piece of scientific research with data that supports the conclusions. Experiments must have been conducted rigorously, with appropriate controls, replication, and sample sizes. The conclusions must be drawn appropriately based on the data presented.

Reviewer #4: Yes

**********

3. Has the statistical analysis been performed appropriately and rigorously?

Reviewer #4: Yes

**********

4. Have the authors made all data underlying the findings in their manuscript fully available?

The PLOS Data policy requires authors to make all data underlying the findings described in their manuscript fully available without restriction, with rare exception (please refer to the Data Availability Statement in the manuscript PDF file). The data should be provided as part of the manuscript or its supporting information, or deposited to a public repository. For example, in addition to summary statistics, the data points behind means, medians and variance measures should be available. If there are restrictions on publicly sharing data—e.g. participant privacy or use of data from a third party—those must be specified.

Reviewer #4: (No Response)

**********

5. Is the manuscript presented in an intelligible fashion and written in standard English?

PLOS ONE does not copyedit accepted manuscripts, so the language in submitted articles must be clear, correct, and unambiguous. Any typographical or grammatical errors should be corrected at revision, so please note any specific errors here.

Reviewer #4: Yes

**********

6. Review Comments to the Author

Please use the space provided to explain your answers to the questions above. You may also include additional comments for the author, including concerns about dual publication, research ethics, or publication ethics. (Please upload your review as an attachment if it exceeds 20,000 characters)

Reviewer #4: Thank you for addressing my questions. I particularly appreciate the addition of the following points “This imbalance suggests that patients randomized to SPP-004 were, on average, more severely affected at baseline. It was a factor that would be expected to bias outcomes toward less improvement rather than favoring an apparent treatment effect” and “Given the small sample size, inter-individual variability, and baseline imbalance, the results should be interpreted with caution and regarded as supportive evidence of a potential disease-modifying effect rather than definitive proof of efficacy. “

I have a couple of minor suggestions/comments:

1. The added text on sample size, heterogeneity and cautious interpretation remains primarily focused on efficacy. The manuscript still conveys a strong statement that the SAEs are unrelated to the study drug. In the absence of a clear explanation for the marked imbalance between SPP-004 and placebo group (64% vs 7%), this difference should be more explicitly and transparently acknowledged in the Results/Discussion section.

2. The supplemental figure 1- B shows the NPMDS total score for placebo arm at 48 weeks to be close to 32 while supplemental table 3 reports it to be 38.9. Please address the discrepancy and update the figure 1-A accordingly, if needed.

**********

7. PLOS authors have the option to publish the peer review history of their article (what does this mean?). If published, this will include your full peer review and any attached files.

If you choose “no”, your identity will remain anonymous but your review may still be made public.

**Do you want your identity to be public for this peer review?**  For information about this choice, including consent withdrawal, please see our Privacy Policy.

Reviewer #4: **Yes:**  Shaifali Saroha

**********

[NOTE: If reviewer comments were submitted as an attachment file, they will be attached to this email and accessible via the submission site. Please log into your account, locate the manuscript record, and check for the action link "View Attachments". If this link does not appear, there are no attachment files.]

To ensure your figures meet our technical requirements, please review our figure guidelines: https://journals.plos.org/plosone/s/figures

You may also use PLOS’s free figure tool, NAAS, to help you prepare publication quality figures: https://journals.plos.org/plosone/s/figures#loc-tools-for-figure-preparation.

NAAS will assess whether your figures meet our technical requirements by comparing each figure against our figure specifications.

# Author comment: Author response to Decision Letter 3

**DOI:** 10.1371/journal.pone.0332283.r006

24 May 2026

RE: PONE-D-25-46225-R2

Authors’ Response to Reviewer #4:

Reviewer’s Comments on the second revised manuscript PONE-D-25-46225-R2

I have a couple of minor suggestions/comments:

1. The added text on sample size, heterogeneity and cautious interpretation remains primarily focused on efficacy. The manuscript still conveys a strong statement that the SAEs are unrelated to the study drug. In the absence of a clear explanation for the marked imbalance between SPP-004 and placebo group (64% vs 7%), this difference should be more explicitly and transparently acknowledged in the Results/Discussion section.

Authors’ Response to Reviewer ��>> The adverse events for the open-label period and the DB-period (SAS) were shown in Table 5, which indicated the marked imbalance between SPP-004 and placebo group (64% vs 7%) as pointed out by Reviewer #4. The patients allocated to SPP-004 by randomization had more advanced conditions with higher NPMDS scores compared to those of Placebo group as shown in Supplemental Table 3 and Supplemental Figure 1B. Therefore, their symptoms associated with the disease itself were observed more often in the patients of SP-004 compared to those of Placebo. Therefore, we concluded the SAE observed were unrelated to SP-004. However, this statement doesn’t sound fair as pointed out by Reviewer #4. We should tone down our statement and now describe as follows.

“The SAEs appeared to be unrelated to the study drug, and the outcome was recovery. The SAE symptoms observed in the safety assessment were considered more likely to be associated with the underlying disease rather than the study drug.”

Accordingly, we revised our descriptions regarding Safety in the Results (Lines 541-543, Lines 546-550) and Discussion (Lines 665-670).

2. The supplemental figure 1- B shows the NPMDS total score for placebo arm at 48 weeks to be close to 32 while supplemental table 3 reports it to be 38.9. Please address the discrepancy and update the figure 1-A accordingly, if needed.

Authors’ Response to Reviewer ��>> We are thankful to the reviewer for pointing out a critical discrepancy of the data shown in Supplemental Figure 1B and Supplemental Table 3. The data shown in Supplemental Table 3 are correct. We inadvertently picked up the incorrect Supplemental Figure 1B from our CSR. We noticed that the data plotted in the previous Supplemental Figure 1B were not adjusted by LOCF. The lower value at week 48 in the previous figure without LOCF is likely attributable to differential dropout in the placebo group, where patients with higher NPMDS scores tended to discontinue earlier, leaving patients with relatively lower scores at later time points.

Therefore, we corrected Supplemental Figure 1B with the data adjusted by LOCF, which were identical to those shown in Supplemental Table 3.

Attachment Submitted filename: Response to Reviewers PONE-D-25-46225-R3.docx

# Peer review: Decision Letter 3

**DOI:** 10.1371/journal.pone.0332283.r007

**Authors:** Bhanwar Lal Puniya

28 May 2026

A phase III double-blind, placebo-controlled, randomized withdrawal trial of 5‑aminolevulinic acid hydrochloride with sodium ferrous citrate for efficacy and safety in patients diagnosed as Leigh syndrome

PONE-D-25-46225R3

Dear Dr. Nakajima,

We’re pleased to inform you that your manuscript has been judged scientifically suitable for publication and will be formally accepted for publication once it meets all outstanding technical requirements.

Within one week, you’ll receive an e-mail detailing the required amendments. When these have been addressed, you’ll receive a formal acceptance letter and your manuscript will be scheduled for publication.

An invoice will be generated when your article is formally accepted. Please note, if your institution has a publishing partnership with PLOS and your article meets the relevant criteria, all or part of your publication costs will be covered. Please make sure your user information is up-to-date by logging into Editorial Manager at Editorial Manager® and clicking the ‘Update My Information' link at the top of the page. For questions related to billing, please contact billing support.

If your institution or institutions have a press office, please notify them about your upcoming paper to help maximize its impact. If they’ll be preparing press materials, please inform our press team as soon as possible -- no later than 48 hours after receiving the formal acceptance. Your manuscript will remain under strict press embargo until 2 pm Eastern Time on the date of publication. For more information, please contact onepress@plos.org.

Kind regards,

Bhanwar Lal Puniya, Ph.D.

Academic Editor

PLOS One

Additional Editor Comments (optional):

Reviewers' comments:

Reviewer's Responses to Questions

**Comments to the Author**

1. If the authors have adequately addressed your comments raised in a previous round of review and you feel that this manuscript is now acceptable for publication, you may indicate that here to bypass the “Comments to the Author” section, enter your conflict of interest statement in the “Confidential to Editor” section, and submit your "Accept" recommendation.

Reviewer #3: All comments have been addressed

**********

2. Is the manuscript technically sound, and do the data support the conclusions?

The manuscript must describe a technically sound piece of scientific research with data that supports the conclusions. Experiments must have been conducted rigorously, with appropriate controls, replication, and sample sizes. The conclusions must be drawn appropriately based on the data presented.

Reviewer #3: (No Response)

**********

3. Has the statistical analysis been performed appropriately and rigorously?

Reviewer #3: (No Response)

**********

4. Have the authors made all data underlying the findings in their manuscript fully available?

The PLOS Data policy requires authors to make all data underlying the findings described in their manuscript fully available without restriction, with rare exception (please refer to the Data Availability Statement in the manuscript PDF file). The data should be provided as part of the manuscript or its supporting information, or deposited to a public repository. For example, in addition to summary statistics, the data points behind means, medians and variance measures should be available. If there are restrictions on publicly sharing data—e.g. participant privacy or use of data from a third party—those must be specified.

Reviewer #3: (No Response)

**********

5. Is the manuscript presented in an intelligible fashion and written in standard English?

PLOS ONE does not copyedit accepted manuscripts, so the language in submitted articles must be clear, correct, and unambiguous. Any typographical or grammatical errors should be corrected at revision, so please note any specific errors here.

Reviewer #3: (No Response)

**********

6. Review Comments to the Author

Please use the space provided to explain your answers to the questions above. You may also include additional comments for the author, including concerns about dual publication, research ethics, or publication ethics. (Please upload your review as an attachment if it exceeds 20,000 characters)

Reviewer #3: (No Response)

**********

7. PLOS authors have the option to publish the peer review history of their article (what does this mean?). If published, this will include your full peer review and any attached files.

If you choose “no”, your identity will remain anonymous but your review may still be made public.

**Do you want your identity to be public for this peer review?**  For information about this choice, including consent withdrawal, please see our Privacy Policy.

Reviewer #3: No

**********

# Editor report: Acceptance letter

**DOI:** 10.1371/journal.pone.0332283.r008

**Authors:** Bhanwar Lal Puniya

PONE-D-25-46225R3

PLOS One

Dear Dr. Nakajima,

I'm pleased to inform you that your manuscript has been deemed suitable for publication in PLOS One. Congratulations! Your manuscript is now being handed over to our production team.

At this stage, our production department will prepare your paper for publication. This includes ensuring the following:

* All references, tables, and figures are properly cited

* All relevant supporting information is included in the manuscript submission,

* There are no issues that prevent the paper from being properly typeset

You will receive further instructions from the production team, including instructions on how to review your proof when it is ready. Please keep in mind that we are working through a large volume of accepted articles, so please give us a few days to review your paper and let you know the next and final steps.

Lastly, if your institution or institutions have a press office, please let them know about your upcoming paper now to help maximize its impact. If they'll be preparing press materials, please inform our press team within the next 48 hours. Your manuscript will remain under strict press embargo until 2 pm Eastern Time on the date of publication. For more information, please contact onepress@plos.org.

You will receive an invoice from PLOS for your publication fee after your manuscript has reached the completed accept phase. If you receive an email requesting payment before acceptance or for any other service, this may be a phishing scheme. Learn how to identify phishing emails and protect your accounts at https://explore.plos.org/phishing.

If we can help with anything else, please email us at customercare@plos.org.

Thank you for submitting your work to PLOS ONE and supporting open access.

Kind regards,

PLOS ONE Editorial Office Staff

on behalf of

Dr. Bhanwar Lal Puniya

Academic Editor

PLOS One
